# The Cyclase-Associated Protein Cap1 Is Important for Proper Regulation of Infection-Related Morphogenesis in *Magnaporthe oryzae*

**DOI:** 10.1371/journal.ppat.1002911

**Published:** 2012-09-06

**Authors:** Xiaoying Zhou, Haifeng Zhang, Guotian Li, Brian Shaw, Jin-Rong Xu

**Affiliations:** 1 Department of Botany and Plant Pathology, Purdue University, West Lafayette, Indiana, United States of America; 2 Purdue-NWAFU Joint Research Center, College of Plant Protection, Northwest A&F University, Yangling, Shanxi, China; 3 Department of Plant Pathology and Microbiology, Texas A&M University, College Station, Texas, United States of America; University of Melbourne, Australia

## Abstract

Surface recognition and penetration are critical steps in the infection cycle of many plant pathogenic fungi. In *Magnaporthe oryzae*, cAMP signaling is involved in surface recognition and pathogenesis. Deletion of the *MAC1* adenylate cyclase gene affected appressorium formation and plant infection. In this study, we used the affinity purification approach to identify proteins that are associated with Mac1 *in vivo*. One of the Mac1-interacting proteins is the adenylate cyclase-associated protein named Cap1. CAP genes are well-conserved in phytopathogenic fungi but none of them have been functionally characterized. Deletion of *CAP1* blocked the effects of a dominant *RAS2* allele and resulted in defects in invasive growth and a reduced intracellular cAMP level. The *Δcap1* mutant was defective in germ tube growth, appressorium formation, and formation of typical blast lesions. Cap1-GFP had an actin-like localization pattern, localizing to the apical regions in vegetative hyphae, at the periphery of developing appressoria, and in circular structures at the base of mature appressoria. Interestingly, Cap1, similar to LifeAct, did not localize to the apical regions in invasive hyphae, suggesting that the apical actin cytoskeleton differs between vegetative and invasive hyphae. Domain deletion analysis indicated that the proline-rich region P2 but not the actin-binding domain (AB) of Cap1 was responsible for its subcellular localization. Nevertheless, the AB domain of Cap1 must be important for its function because *CAP1* ^ΔAB^ only partially rescued the *Δcap1* mutant. Furthermore, exogenous cAMP induced the formation of appressorium-like structures in non-germinated conidia in *CAP1* ^ΔAB^ transformants. This novel observation suggested that AB domain deletion may result in overstimulation of appressorium formation by cAMP treatment. Overall, our results indicated that *CAP1* is important for the activation of adenylate cyclase, appressorium morphogenesis, and plant infection in *M. oryzae*. *CAP1* may also play a role in feedback inhibition of Ras2 signaling when Pmk1 is activated.

## Author Summary

In *Magnaporthe oryzae*, cAMP signaling is known to play an important role in surface recognition and plant penetration. The Mac1 adenylate cyclase is essential for plant infection. To better understand Mac1 activation mechanisms, in this study we used the affinity purification approach to identify proteins that are associated with Mac1 *in vivo*. One of the Mac1-interacting protein is the adenylate cyclase associated protein (CAP) encoded by the *CAP1* gene. Results from our study indicated that Cap1 is important for Mac1 activation and plant infection in *M. oryzae*. The *Δcap1* mutant was defective in germ tube growth and appressorium formation and failed to cause typical blast lesions. Like LifeAct, Cap1 localized to apical patches in vegetative hyphae but not in invasive hyphae. The P2 proline-rich region was important for Cap1 localization but the actin-binding domain played a role in feedback inhibition of Ras signaling. To our knowledge, functional characterization of CAP genes has not been reported in filamentous fungi. Our results indicate that *CAP1* is important for regulating adenylate cyclase activities, appressorium morphogenesis, and plant infection. Further characterization of *CAP1* will be important to better understand the interaction between cAMP signaling and the *PMK1* pathway in *M. oryzae*.

## Introduction

*Magnaporthe oryzae*, a heterothallic ascomycete, is the causal agent of rice blast, which is one of the most destructive diseases of rice in the world. It infects the rice plant in a manner typical of many foliar pathogens. Germ tubes differentiate into specialized infection structures called appressoria. The fungus generates turgor pressure as high as 8 MPa in mature appressoria for plant penetration [1]. In *M. oryzae*, appressorium formation can be induced by attachment to hydrophobic surfaces that mimic the rice leaf surface. On hydrophilic surfaces, treatments with cAMP, cutin monomers, or primary alcohols induce the formation of melanized appressoria. To date, at least three putative sensor genes, *PTH11*, *CBP1*, and *MSB2*, have been reported to be involved in the recognition of physical and chemical signals of plant leaves [2], [3], [4]. In *M. oryzae*, cyclic AMP (cAMP) is known to be the intracellular secondary messenger. Molecular studies have further confirmed the role of the cAMP-PKA pathway in surface recognition [5], [6], [7]. The *CPKA* gene encoding a catalytic subunit of PKA is required for normal appressorium formation and plant infection [8], [9]. The Mac1 adenylate cyclase responsible for the synthesis of intracellular cAMP is required for appressorium formation and pathogenesis [5], [6]. The surface attachment and recognition signals must be somehow relayed from the surface sensors to the activation of *MAC1*, which may involve changes in the actin cytoskeleton because germ tube tip deformation is the initiation stage of appressorium formation. In mammalian cells, adenylyl cyclase AC8 orchestrates its downstream activity to yield a high output signaling by its association with actin cytoskeleton [10].

Although it is dispensable for surface recognition, the Pmk1 MAP kinase pathway is known to regulate late stages of appressorium formation, appressorial penetration, and invasive growth in *M. oryzae* [11], [12]. In a number of plant pathogenic fungi, the orthologous MAPK cascade is conserved for the regulation of various infection or developmental processes [13]. In *M. oryzae*, the surface recognition signals must be conveyed from cAMP signaling to the Pmk1 MAPK pathway for appressorium formation and plant penetration, but the exact molecular mechanism is not clear. The adaptor protein of the Pmk1 MAPK cascade, Mst50, is known to interact with both Mst11 and Mst7. It also interacts with the two Ras proteins in *M. oryzae*, Ras1 and Ras2, in yeast two-hybrids assays. Ras2 is likely involved in the activation of both cAMP-PKA and Pmk1 MAP kinase pathways [12], [14]. Transformants expressing the dominant active allele of *RAS2* form melanized appressoria on both hydrophilic and hydrophobic surfaces.

In the budding yeast *Saccharomyces cerevisiae*, Ras2 regulates the activation of adenylyl cyclase (AC). The yeast Cyr1 adenylyl cyclase is associated with a 70-kDa cyclase-associated protein (CAP) that was identified in a RAS-responsive adenylyl cyclase complex [15]. The CAP gene also was identified as *SRV2* in a genetic screen for suppressors of a constitutive active *RAS2* ^G19V^ allele [16], [17]. The yeast Srv2 protein has two distinct functional domains. Whereas the N-terminal adenylyl cyclase-binding (ACB) domain is sufficient for full cellular responsiveness to Ras2 and Cyr1, the C-terminal actin-binding domain interacts with actin monomers. Loss of the C-terminal region of Srv2 caused morphological and nutritional defects that are not related to adenylyl cyclase or Ras2 activity. The C-terminal region of Srv2 plays a critical role in binding to G-actin *in vivo* and directing actin organization and polarized cell growth. However, recent studies suggested the N-terminus is equally important for Srv2 in driving actin turnover [18], [19], [20]. The CAP proteins are well conserved in eukaryotic organisms. Orthologs of Srv2 have been characterized in *Schizosaccharomyces pombe*, *Candida albicans*, *Cryptococus neoformans*, and *Ustilago maydis* [17], [21], [22], [23]. In *C. albicans*, *CAP1* is involved in initiation of the transition of yeast cells to hyphal growth. It also interacts with Ras and AC proteins to regulate the intracellular cAMP level. The *cap1/cap1* mutant was reduced in virulence in a mouse model system and was defective in the yeast-hyphae transition, which can be stimulated by cAMP treatments [24], [25]. In *C. neoformans*, Cap1 is a positive regulator of Cac1 and is required for the cAMP-mediated capsule formation and virulence [23]. The *CAP1* ortholog in *U. maydis*, as an additional component of the cAMP/PKA signaling pathway, interacts with adenylate cyclase Uac1 and is important for morphogenesis and pathogenesis [26].

Although the cAMP signaling pathway has been shown to play important roles in various plant pathogenic fungi, to date no CAP genes have been functionally studied in filamentous ascomycetes. In this study, we identified and characterized the *CAP1* (for cyclase-associated protein 1) gene in *M. oryzae*. Cap1 interacts with Mac1 in yeast two-hybrid and co-immunoprecipitation (co-IP) assays. It was found to be important for the activation of Mac1 and invasive growth. The Δ*cap1* mutant was defective in appressorium formation, germ tube growth, and plant infection. Like LifeAct [27], Cap1 localized to apical patches in vegetative hyphae but not in invasive hyphae, indicating that invasive hyphae may lack a typical actin cytoskeleton at the tip. Domain deletion analysis revealed that the AB domain of Cap1 was dispensable for its subcellular localization but plays a role in the proper regulation of appressorium formation and full virulence. Overall, our results indicate that *CAP1* is important for the activation of adenylate cyclase, appressorium morphogenesis, and plant infection in *M. oryzae*.

## Results

### Identification of proteins associated with Mac1 *in vivo*

To identify genes interacting with *MAC1* in *M. oryzae*, we generated the 3×FLAG knock-in construct and transformed it into the wild-type stain 70-15 (Table 1). In the resulting transformant MFG1, a 242-kDa band of expected size of Mac1-3×FLAG fusion was detected by an anti-FLAG antibody (Fig. S1A). For affinity purification, proteins bound to anti-FLAG M2 beads were eluted and digested with trypsin. Proteins that co-purified with Mac1-3×FLAG were identified by mass spectrometry (MS) analysis as described [28]. One of the Mac1-interacting genes (Table 2) is MGG_01722.6 that encodes a protein highly similar to Srv2 in *S. cerevisiae* [29]. Srv2 is a cyclase-associated protein (CAP) involved in the Ras/cAMP pathway. Other proteins that co-immunoprecipitated with Mac1 included several components of the protein phosphatase PP2A and a number of hypothetical proteins unique to *M. oryzae* (Table 2). In mammalian cells, catalytic and regulatory subunits of PP2A also were co-immunoprecipitated with the N-terminal region of adenylyl cyclase 8 [30].

**Table 1:** Wild-type and mutant strains of *Magnaporthe oryzae* used in this study.

Strain	Genotype description	Reference
70-15	Wild-type (*MAT1-1*, *AVR-Pita*)	Chao & Ellingboe, 1991
Guy11	Wild-type (*MAT1-2*, *AVR-Co39*)	Leung et al. 1988
Ku80	*MgKu80* deletion mutant of Guy11	Villalba et al., 2008
nn78	*Δpmk1* mutant	Xu & Hamer, 1996
HC82	*Δcap1* deletion mutant of Ku80	This study
HC83	*Δcap1* deletion mutant of Ku80	This study
HF12	*Δcap1* mutant of Guy11	This study
HF13	HF12 transformed with *CAP1*	This study
HF14	HF12 transformed with *CAP1^Δ^* ^AC^	This study
HF15	HF12 transformed with *CAP1^Δ^* ^PR^	This study
HC10	*CAP1* ^ΔAB^ (Δ375–534) knock-in transformant of Ku80	This study
CH07	*CAP1* complementation transformant (*Δcap1*/*CAP1*)	This study
CH10	*CAP1* ^ΔAB^/*CAP1* complementation transformant	This study
HZ90	*RAS2* ^DA^ transformant of Guy11	This study
XY22	*RAS2* ^DA^ transformant of the Δ*cap1* deletion mutant	This study
XY60	*CAP1*-3×FLAG transformant of the Δ*cap1* mutant	This study
XY61	*CAP1*-GFP transformant of the Δ*cap1* mutant	This study
XY109	*CAP1* ^ΔACB^–3×FLAG transformant the Δ*cap1* mutant	This study
XY94	*CAP1* ^ΔACB^–GFP transformant of the Δ*cap1* mutant	This study
XY110	*CAP1* ^ΔAB^–3×FLAG transformant the Δ*cap1* mutant	This study
XY105	*CAP1* ^ΔAB^–GFP transformant of the Δ*cap1* mutant	This study
XY95	*CAP1* ^ΔP1^-GFP transformant of the Δ*cap1* mutant	This study
XY244	*CAP1* ^ΔP2^-GFP transformant of the Δ*cap1* mutant	This study
MCF12	*MAC1*-3×FLAG transformant of 70-15	This study
CMT8	*CAP1*-GFP and *MAC1* ^CT^-3×FLAG transformant of 70-15	This study
DCN22	*CAP1* ^ΔACB^-GFP and *MAC1* ^CT^-3×FLAG transformant of 70-15	This study
CMB14	*CAP1-*NYFP and *MAC1* ^CT^-CYFP transformant of 70-15	This study
MFG1	*MAC1*-3×FLAG knock-in transformant of Guy11	This study
CGS	Transformant of mutant HC10 expressing *GAS2*-GFP	This study
HC1-6	*CAP1* ^ΔAB^ *Δpmk1* mutant	This study
HC1-39	*CAP1* ^ΔAB^ *Δpmk1* mutant	This study
MC20	LifeAct-GFP and *CAP1*-mCherry transformant of Guy11	This study
LA31	LifeAct-GFP transformant of Guy11	This study
XY001	*CAP1* ^ΔP2^-GFP transformant of the Δ*cap1* deletion mutant	This study
XY002	*CAP1* ^ΔACB^ (Δ2–166) knock-in transformant of Ku80	This study
XY111	*CAP1* ^ΔACB^ (Δ2–166) knock-in transformant of Ku80	This study

**Table 2:** Putative Mac1-interacting genes identified by affinity purification.

Gene ID	Predicted function
MGG_01722.6	Adenylate cyclase-associated protein, orthologous to yeast *SRV2*
MGG_05671.6	Regulatory subunit A of PP2A, orthologous to yeast *TPD3*
MGG_05637.6	Regulatory subunit B of PP2A, orthologous to yeast *CDC55*
MGG_06099.6	Catalytic subunit A of PP2A, orthologous to yeast *PPH22*
MGG_12130.6	Regulatory subunit B of PP2A, orthologous to *RTS1*
MGG_09935.6	Activator of the phosphotyrosyl phosphatase activity of PP2A, orthologous to yeast *RRD2*
MGG_12118.6	Serine/threonine-protein kinase, orthologous to *KIC1*
MGG_06482.6	MAP kinase kinase, orthologous to *MKK1*
MGG_02846.6	Orthologous to yeast *SRB8*
MGG_03174.6	Orthologous to yeast *LIN1*, with a GYF domain
MGG_03232.6	Orthologous to yeast *SOG2*, with a leucin rich repeat
MGG_03942.6	Orthologous to yeast *BOP3*
MGG_04190.6	Orthologous to yeast *SRP68*
MGG_07112.6	Hypothetical protein unique to *M. oryzae*
MGG_01805.6	Hypothetical protein unique to *M. oryzae*
MGG_09997.6	Hypothetical protein unique to *M. oryzae*
MGG_10480.6	Hypothetical protein unique to *M. oryzae*
MGG_10588.6	Serine-threonine kinase receptor-associated protein
MGG_04321.6	Conserved hypothetical protein in filamentous fungi
MGG_11259.6	Conserved hypothetical protein in filamentous fungi
MGG_08391.6	Conserved hypothetical protein in filamentous fungi

### Cap1 physically interacts with Mac1

**Figure 1:**
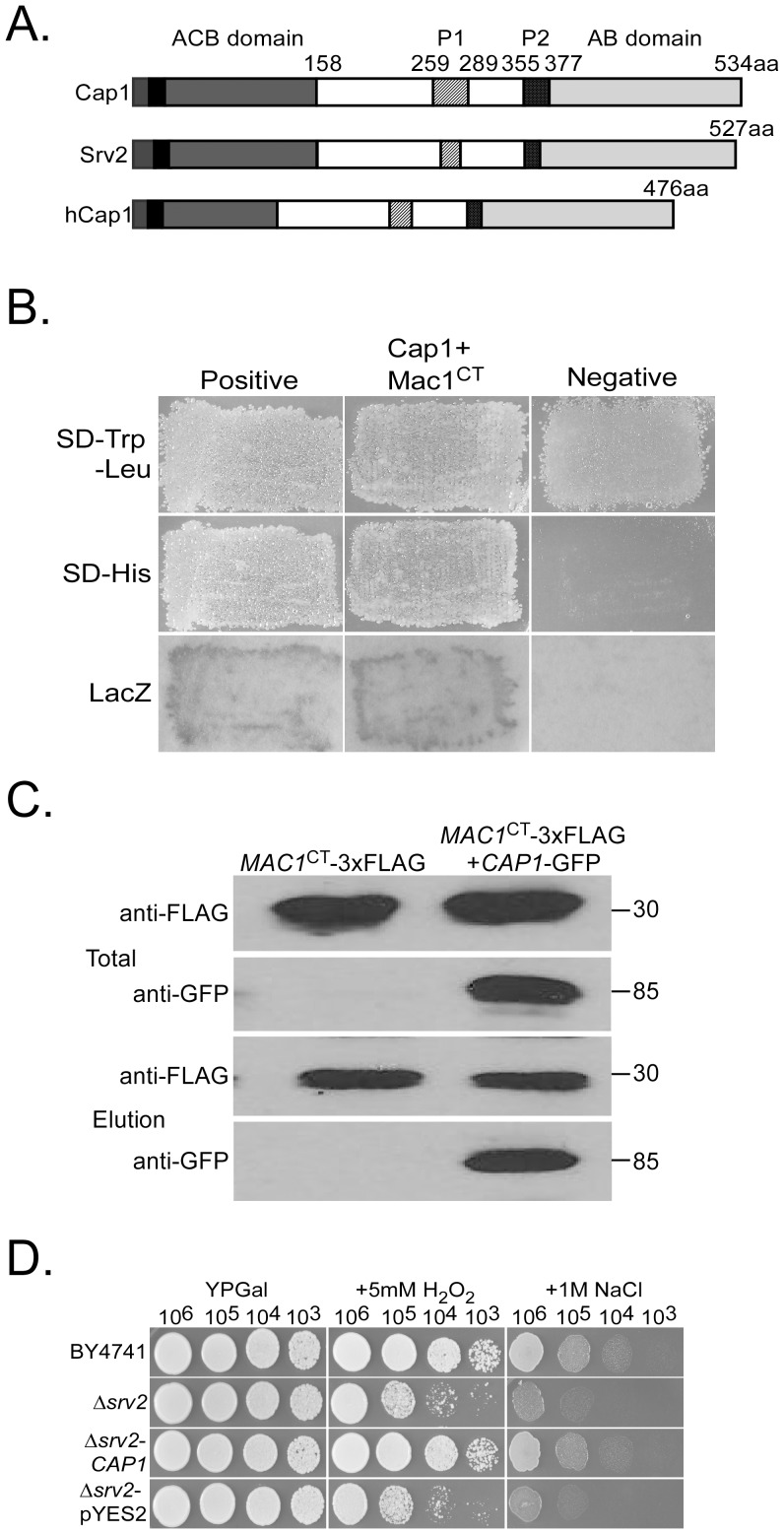
Assays for the interaction of *CAP1* with *MAC1* and its function in yeast. **A.** The domain structure of *M. oryzae* Cap1, *S. cerevisiae* Srv2, and human Cap1(hCap1). ACB, AC-binding domain*;* P1 and P2, proline-rich regions; AB; actin-binding domain. **B.** Yeast transformants expressing the *CAP1* prey and *MAC1* ^CT^ bait constructs were assayed for growth on SD-Trp-Leu and SD-His plates and ß-galactosidase activities (LacZ). The Mst11-Mst50 and Pmk1-Mst50 interactions were the positive and negative controls. **C.** Co-IP assays. Western blots of total proteins and proteins eluted from anti-FLAG M2 beads from transformant CMT (*CAP1*-GFP and *MAC1* ^CT^-3×FLAG) and transformant MCF (*MAC1* ^CT^-3×FLAG) were detected with anti-FLAG or anti-GFP antibodies. **D.** Yeast cells (10^3^ to 10^6^ cells/ml) of BY4741, Δ*srv2* mutant, and Δ*srv2* -*CAP1* or Δ*srv2* -pYES2 transformants were assayed for growth on YPGal (galactose) plates with or without 5 mM H_2_O_2_ or 1 M NaCl.

MGG_01722.6 was named *CAP1* (for cyclase- associated protein 1) and selected for further characterization in this study. It has an AC-binding (ACB) domain (2–166 aa), two proline-rich regions P1 (257–290 aa) and P2 (355–377 aa), and a C-terminal actin-binding (AB) domain (378–534 aa) (Fig. 1A). In *S. cerevisiae*, Srv2 interacts with the C-terminal alpha helix region of Cyr1 adenylyl cyclase *via* its tandem repeats of the heptad motif aXXaXXX [31], which are conserved in *CAP1* and its orthologs in other fungi (Fig. S1B). In *M. oryzae*, Cap1 interacted with the C-terminal region of Mac1 (1926–2160 aa, Mac1^CT^) in yeast two-hybrid assays (Fig. 1B), suggesting the direct association between Cap1 and Mac1.

To confirm their interaction, the *MAC1* ^CT^-3×FLAG and *CAP1*-GFP fusion constructs were co-transformed into protoplasts of strain 70-15. One of the resulting transformant was CMT8 (Table 1). In western blot analysis with total proteins isolated from transformant CMT8, the anti-FLAG and anti-GFP antibodies detected a 30-kDa and a 85-kDa band, respectively. In proteins eluted from anti-FLAG M2 beads, the 85-kDa Cap1-GFP band was detected with an anti-GFP antibody in transformant CMT8 but not in transformant MCF12 (Fig. 1C). Transformant MCF12 expressing the *MAC1* ^CT^-3×FLAG construct only (Table 1) was the negative control. These results indicate that Cap1 interacts with Mac1 in *M. oryzae*.

To determine whether the N-terminal ACB domain is important for the interaction of Cap1 with Mac1, the *CAP1^Δ^* ^ACB^-GFP and *MAC1* ^CT^-3×FLAG constructs were co-transformed into the wild type strain 70-15. In the resulting transformants, both Cap1*^Δ^* ^ACB^-GFP and Mac1^CT^-3×FLAG were expressed. In proteins eluted from anti-FLAG M2 beads, the 30-kDa Mac1^CT^-3×FLAG band was detected by an anti-FLAG antibody. However, we failed to detect the 67-kDa Cap1*^Δ^* ^ACB^-GFP band (Fig. S2), indicating that deletion of the ACB domain eliminated the interaction between Cap1 and Mac1. These results indicate that the N-terminal ACB domain of Cap1 is essential for its interaction with Mac1.

**Figure 2:**
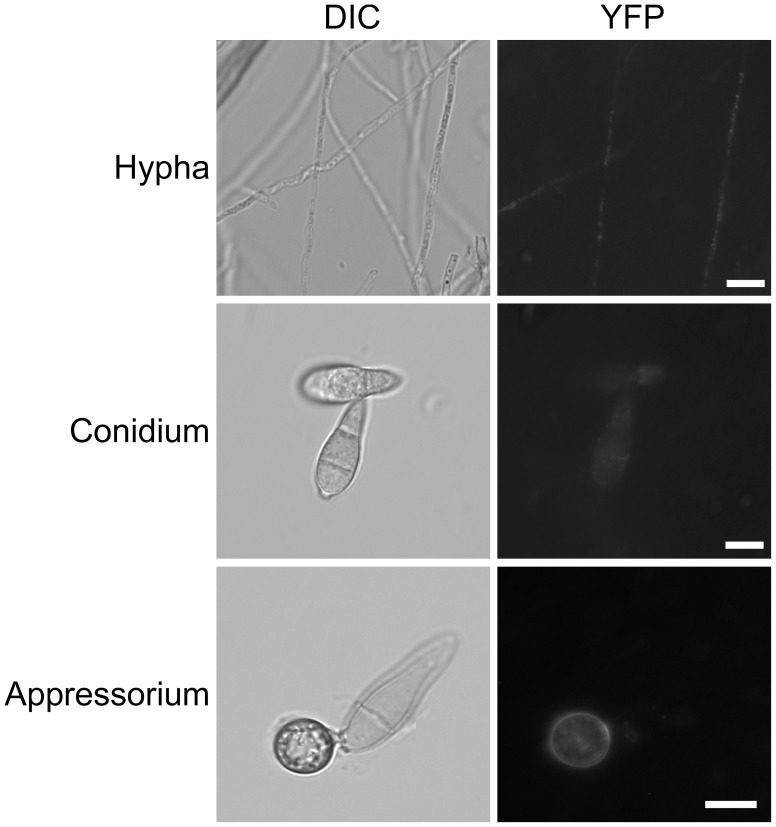
BiFC assays for the Cap1-Mac1 interaction. Vegetative hyphae, conidia, and appressoria of transformant CMB14 expressing the *CAP1*-NYFP and *MAC1* ^CT^-CYFP constructs were examined by DIC and epifluorescence microscopy. Bar = 10 µm.

To further characterize the Cap1-Mac1 interaction *in vivo*, the *CAP1*-NYFP and *MAC1* ^CT^-CYFP fusion constructs were generated and transformed into protoplasts of the wild type strain 70-15 for bimolecular fluorescence complementation (BiFC) assays. In the resulting transformant CMB14 (Table 1), weak YFP signals were observed in the cytoplasm of vegetative hyphae and conidia. In appressoria, stronger YFP signals were observed in the cytoplasm and on cytoplasm membrane (Fig. 2). The presence of YFP signals on the cytoplasm membrane is consistent with the membrane association of adenylyl cyclase in mammalian cells, yeast, and other organisms [32], [33], [34]. These results confirmed that Cap1 interacts with Mac1 *in vivo*. The interaction between Cap1 and Mac1 may be weak during vegetative growth but enhanced during appressorium formation.

### *CAP1* rescues the defects of the *srv2* mutant

The amino acid sequence of *M. oryzae* Cap1 shares 40% identity with yeast Srv2. To test whether *CAP1* is functional in *S. cerevisiae*, the *CAP1* ORF was cloned into pYES2 and transformed into the *srv2* mutant, which is hypersensitive to osmotic and oxidative stresses [35]. The resulting Trp^+^ yeast transformants containing the pYES2-*CAP1* construct grew better than the original *srv2* mutant on medium containing 5 mM H_2_O_2_ or 1 M NaCl (Fig. 1D). In contrast, strains transformed with the empty pYES2 vector were as sensitive as the *srv2* mutant to hyperosmotic or oxidative stresses (Fig. 1D). Thus, when it was expressed in yeast, *CAP1* could suppress the defects of the *srv2* mutant in stress responses.

### Vegetative growth and conidiation are reduced in the *Δcap1* mutant

To characterize its function in *M. oryzae*, the *CAP1* gene replacement construct (Fig. S3A) was generated by ligation PCR [36] and transformed into protoplasts of Ku80. Seven putative *Δcap1* mutants were identified and confirmed by Southern blot analysis (Fig. S3B). All seven mutants had the same phenotype, although only mutant HC83 (Table 1) is described below for detailed analysis. The *Δcap1* mutant was reduced in vegetative growth (Fig. S3C) and conidiation (Table 3). We also generated the *cap1* deletion mutant in the Guy11 background. The resulting *Δcap1* mutant HF12 (Table 1) had similar defects as mutant HC83.

**Table 3:** Phenotypes of the *cap1* mutant in growth, conidiation, and plant infection.

Strain	Growth rate (mm/day)^a^	Conidiation (×10^5^ spores/plate)	Appressoria/germ tube^b^ (%)	Lesions/5 cm leaf tip^c^	Appressorium penetration^d^ (%)
Ku80 (wt)	3.3±0.1	296.3±28.8	96.2±1.8 A*	51.3±4.5 A	68.4±5.4 A
HC83 (Δ*cap1*)	1.7±0.2	112.1±13.4	51.3±1.7 C	9.4±2.1 D	15.2±3.2 D
CH07 (*Δcap1/CAP1*)	3.3±0.1	310.5±21.2	95.0±2.0 A	47.3±5.2 A	62.5±4.8 A
HC10 (*CAP1* ^ΔAB^)	2.7±0.1	259.7±5.9	91.5±1.4 A	31.5±1.3 B	49.6±5.1 B
XY111 (*CAP1* ^ΔACB^)	1.8±0.1	153.6±11.6	59.6±2.7 C	11.5±2.9 D	NA
XY244 (*CAP1* ^ΔP2)^	3.1±0.2	307.3±25.3	69.0±1.2 C	17.3±4.3 C	33.2±1.9 C

a Growth rate and conidiation were measured on complete medium (CM). Mean and standard deviation were calculated with results from three replicates.

b Appressorium formation is expressed as the percentage of germ tubes producing appressorium.

c Lesion formation was examined on infected rice leaves 7 days post-inoculation.

Means and SD values were calculated from at least three independent experiments.

d Appressorium penetration was assayed on onion epidermis at 48 h after inoculation.

* The same letter indicated there was no significant difference. Different letters were used to mark statistically significant differences (P = 0.05).

We then cloned the full length *CAP1* gene into pYK11 as pXY63 and transformed it into the *Δcap1* mutant HC83. The resulting *Δcap1/CAP1* transformant CH07 (Table 1) was normal in growth and conidiation (Table 3). It also was normal in appressorium formation and plant infection (see below), indicating that the reintroduction of the wild-type *CAP1* allele fully complemented the defects of the *Δcap1* mutant.

### Abnormal germ tube growth and appressorium morphology in the *Δcap1* mutant

**Figure 3:**
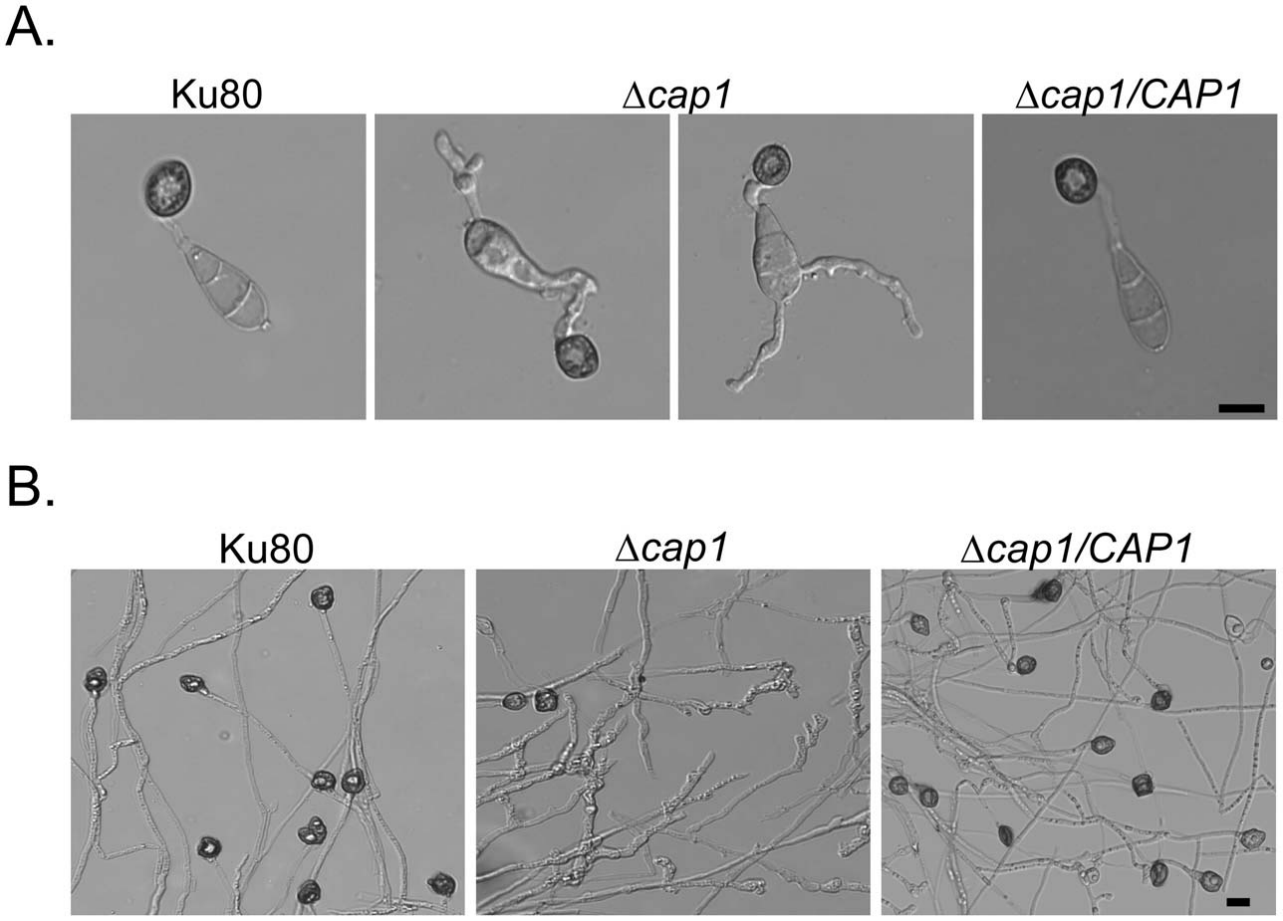
Appressorium formation assays on hydrophobic surfaces. **A.** Appressoria formed by Ku80, Δ*cap1* mutant HC83, and Δ*cap1/CAP1* transformant CH07 on the hydrophobic surface of GelBond membranes at 24 h. **B.** Appressoria formed by hyphal tips on hydrophobic surfaces after incubation for 48 h. Bar = 10 µm.

On hydrophilic surfaces, like the wild type, the *Δcap1* mutant failed to form appressoria. It still formed melanized appressoria on hydrophobic surfaces but the efficiency of appressorium formation per germ tube was significantly reduced (Table 3). In addition, germ tubes of the *Δcap1* mutant had abnormal morphology and growth patterns. Besides producing branching germ tubes with uneven width (Fig. 3A), 54.0±1.9% of the conidium compartments produced more than one germ tubes in the *Δcap1* mutant. Subapical swollen bodies similar to those observed in the *pmk1* mutant [37] were often formed by germ tubes of the *Δcap1* mutant (Fig. 3A). We also assayed appressorium formation with hyphal tips on hydrophobic surfaces. Whereas less than 10% of the hyphal tips in the *Δcap1* mutant developed appressoria, majority of hyphal tips formed appressoria by 48 h in Ku80 (Fig. 3B). Most of the *Δcap1* hyphae that attached to hydrophobic surfaces formed irregular apical and intercalary swellings (Fig. 3B), further indicating the importance of *CAP1* in appressorium formation.

### Deletion of *CAP1* suppresses the effects of *RAS2* ^DA^ on appressorium formation

**Figure 4:**
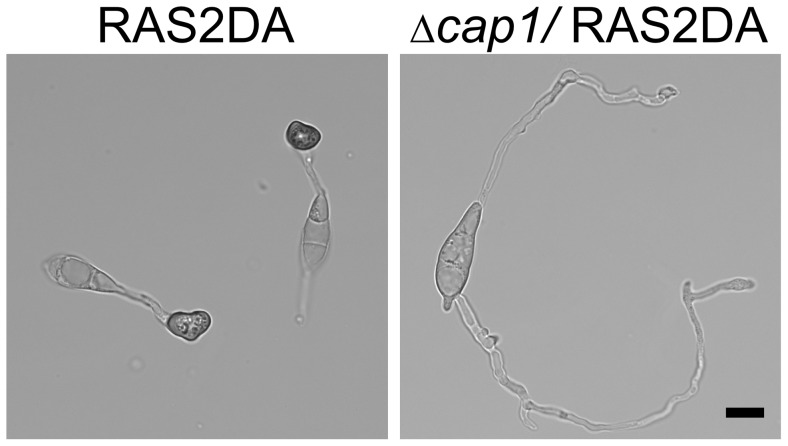
Deletion of *CAP1* suppressed the formation of appressoria on hydrophilic surfaces in the *RAS2* ^DA^ transformant. Conidia of the *RAS2* ^DA^ and the *cap1 RAS2* ^DA^ transformants were incubated on the hydrophilic surface of GelBond membranes. After incubation for 24 h, melanized appressoria were formed by the *RAS2* ^DA^ transformant but not the *cap1 RAS2* ^DA^ transformant.

Expressing the dominant active allele *RAS2* ^DA^ in the wild type resulted in the over-activation of the cAMP and Pmk1 pathways and formation of appressoria on both hydrophobic and hydrophilic surfaces [38]. Because *SRV2* was first identified as a suppressor of the dominant active mutation of Ras, we introduced the *RAS2* ^DA^ allele into the Δ*cap1* mutant. The resulting Δ*cap1 RAS2* ^DA^ transformant XY22 (Table 1) failed to form appressoria on hydrophilic surfaces (Fig. 4). In contrast, the *RAS2* ^DA^ transformant of wild type Guy11 [14], [38] formed appressoria on hydrophilic surfaces. On hydrophobic surfaces, the Δ*cap1 RAS2* ^DA^ transformant, similar to the original Δ*cap1* mutant, formed melanized appressoria.

### The *Δcap1* mutant has a reduced intracellular cAMP level

**Figure 5:**
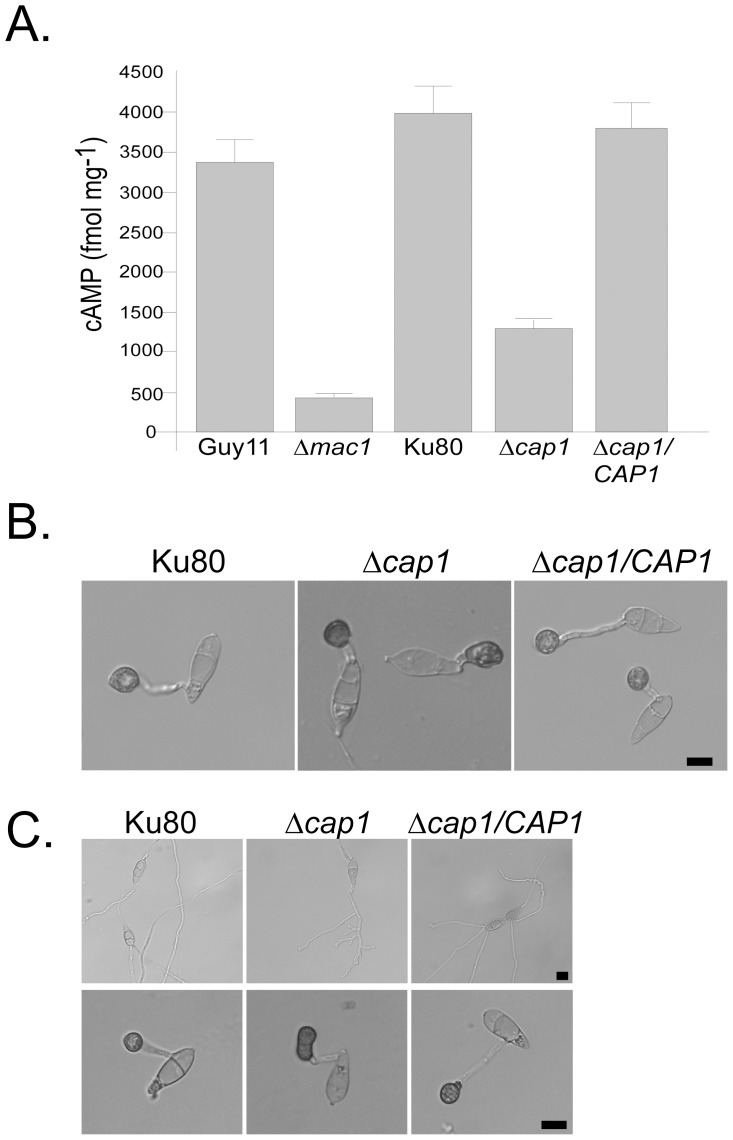
*CAP1* functions in the cAMP signaling pathway. **A.** The intracellular cAMP level in Guy11, Δ*mac1* mutant, Ku80, Δ*cap1* mutant HC83, and Δ*cap1/CAP1* transformant CH07. Mean and standard deviation were calculated with results from three independent biological replicates. **B.** Appressorium formation assays with Ku80, HC83, and CH07 in the presence of 5 mM cAMP on the hydrophobic surface of GelBond membranes. **C.** Appressorium formation assays on the hydrophilic surface of GelBond membranes in the absence (upper panel) or presence (lower panel) of 5 mM cAMP. Bar = 10 µm.

To determine whether *CAP1* is functionally related to the activation of *MAC1* in *M. oryzae*, we assayed the intracellular cAMP content in vegetative hyphae. The *Δcap1* mutant had a higher cAMP level than the *Δmac1* mutant. However, its intracellular cAMP level was significantly reduced in comparison to that of strain Ku80 or Guy11 (Fig. 5A). *MAC1* is the sole adenylate cyclase gene in *M. oryzae*. Detection of cAMP in the *Δmac1* mutant likely was from the background. These results indicate that Cap1 is not essential for Mac1 activation but plays an important role in the full activation of Mac1 in *M. oryzae*.

### Exogenous cAMP partially suppresses the defects of the *Δcap1* mutant

Because the *Δcap1* mutant had a reduced intracellular cAMP level, we assayed the effect of exogenous cAMP. On hydrophobic surfaces, cAMP treatment suppressed germ tube branching and growth defects of the *Δcap1* mutant (Fig. 5B). Melanized appressoria were formed on short germ tubes in the presence of 5 mM cAMP.

On hydrophilic surfaces, 43.0±2.3% of the mutant germ tubes formed appressoria by 24 h in the presence of 5 mM cAMP. Under the same conditions, 80.1±3.7% of the wild-type germ tubes formed appressoria. In addition, the majority of appressoria induced by cAMP treatment in the *Δcap1* mutant had abnormal morphology (Fig. 5C). These results indicate that the *Δcap1* mutant still responds to cAMP treatment. *CAP1* must function upstream of cAMP by regulating the activation of Mac1. However, the defects of the *Δcap1* mutant in appressorium morphology suggest that *CAP1* also plays a role in the proper regulation of responses to exogenous cAMP for appressorium morphogenesis.

### The *Δcap1* mutant is significantly reduced in virulence

**Figure 6:**
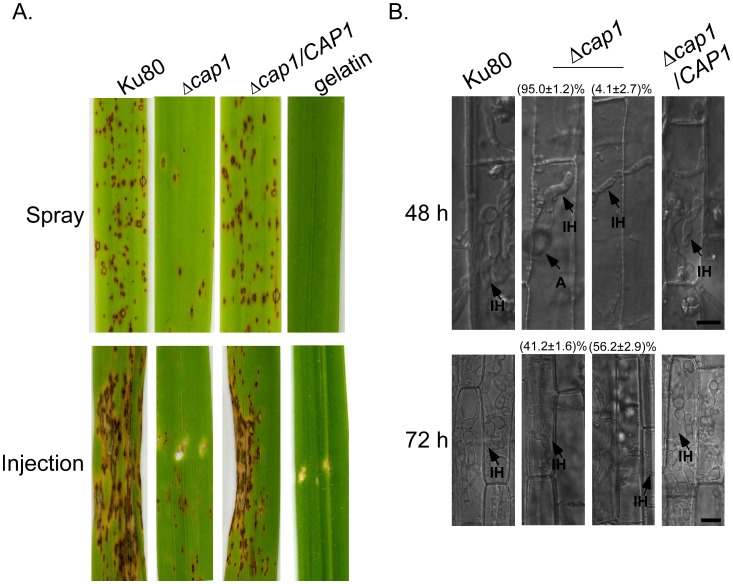
Infection and penetration assays with the Δ*cap1* mutant. **A**. Leaves of two-week-old rice seedlings were sprayed or injected with conidial suspensions of Ku80, Δ*cap1* mutant, and complemented strain Δ*cap1/CAP1*. Inoculation with 0.25% gelatin was used as the negative control. Typical leaves were photographed 7 dpi. **B**. Penetration assays with rice leaf sheaths. Invasive hyphae formed by Ku80, Δ*cap1* mutant, and complemented strain Δ*cap1/CAP1* in plant cells were examined 48 and 72 hpi. A, appressorium; IH, invasive hyphae. Bar = 10 µm.

In spray infection assays with two-week-old rice seedlings of cultivar CO-39, numerous blast lesions were observed on leaves sprayed with Ku80 or the complemented transformant at 7 dpi (Fig. 6A). Under the same conditions, fewer and smaller lesions were observed on leaves sprayed with the *Δcap1* mutant (Fig. 6A). The number of lesions caused by the *Δcap1* mutant was reduced about 6-fold in comparison with Ku80 (Table 3). More importantly, typical blast lesions with extensive necrotic zones were rarely or not observed on leaves inoculated with the *Δcap1* mutant (Fig. 6A). On wound-inoculated leaves, the *Δcap1* mutant also caused fewer and smaller lesions outside the wound sites than Ku80 and the complemented transformant (Fig. 6A). Even at the wounding sites, the *Δcap1* mutant caused no or only limited necrosis. These results indicate that the *CAP1* gene plays a critical role in lesion development and spread of blast infection.

### *CAP1* is important for appressorial penetration and invasive growth

To further characterize the defects of the *Δcap1* mutant in plant infection, we conducted penetration assays with rice leaf sheaths. Penetration by appressoria formed by the *Δcap1* mutant was reduced over 4 folds. Even for those appressoria that successfully penetrated, invasive hyphae formed by the *Δcap1* mutant inside plant cells were narrower than those of Ku80. At 48 hpi, approximately 15% of the appressoria penetrated into rice leaf sheath cells and had limited growth of unbranched, non-bulbous invasive hyphae. Most of invasive hyphae were restricted to the penetrated cells. Under the same conditions, invasive hyphae formed by Ku80 grew extensively in the penetrated and neighboring cells (Fig. 6B). Even at 72 hpi, invasive hyphae of the *Δcap1* mutant were restricted to the initial penetrated cells at 41.2±1.6% of penetration sites examined (Fig. 6B). At the sites where invasive hyphae had spread into neighboring plant cells (56.2±2.9%), the *Δcap1* mutant still had limited growth and rarely branched (Fig. 6B). These data indicate that the *Δcap1* mutant was defective in invasive growth, which may be directly responsible for the reduced lesion size or limited necrotic zones. Failure to develop branching and bulbous invasive hyphae indicates that switching to pseudohyphal growth from primary invasive hyphae may be blocked or significantly attenuated in the *Δcap1* mutant.

### The expression and subcellular localization of Cap1-GFP proteins

**Figure 7:**
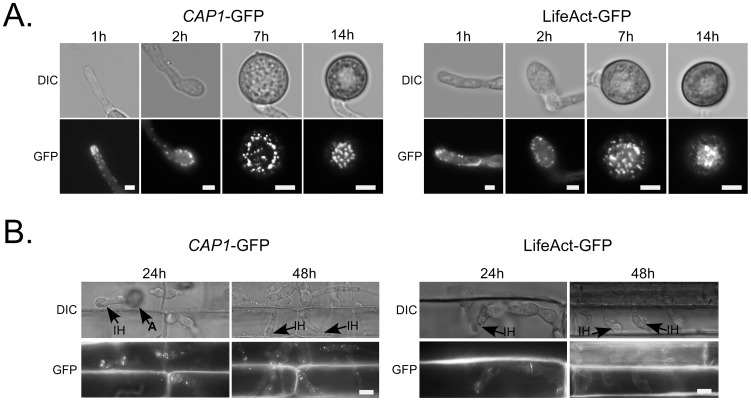
Subcellular localization of Cap1 and LifeAct in *M. oryzae*. **A**. Germ tubes (1 h), developing appressoria (2 h), young appressoria (7 h), and mature appressoria (14 h) of the *CAP1*-GFP (left, Δ*cap1/CAP1*-GFP) and LifeAct-GFP transformants were examined by DIC or epifluorescence microscopy. Bar = 5 µm. **B**. Invasive hyphae produced by the *CAP1*-GFP (left, Δ*cap1/CAP1*-GFP) and LifeAct-GFP transformants in rice leaf sheath were examined 24 or 48 hpi. A, appressorium; IH, invasive hyphae. Bar = 10 µm.

A *CAP1*-GFP fusion construct was generated and transformed into the *Δcap1* mutant. In the resulting transformant XY61 (Table 1), GFP signals were mainly in the apical regions of hyphae or germ tubes (Fig. 7A). The localization pattern of Cap1-GFP was similar to that of actin patches in *A. nidulans* [39]. In developing appressoria, GFP signals were distributed in the peripherial regions (Fig. 7A). However, in mature appressoria, Cap1-GFP fusion proteins were mainly localized in globular structures in the appressorium pore area (Fig. 7A; Video. S1). The transition of Cap1-GFP localization from the periphery to the base of the appressoria may be related to actin cytoskeleton rearrangement that occurs during appressorium morphogenesis. Because CAP proteins bind to G-actin, the globular structures at the base of appressoria may serve as the deposits of G-actins prior to penetration.

**Figure 8:**
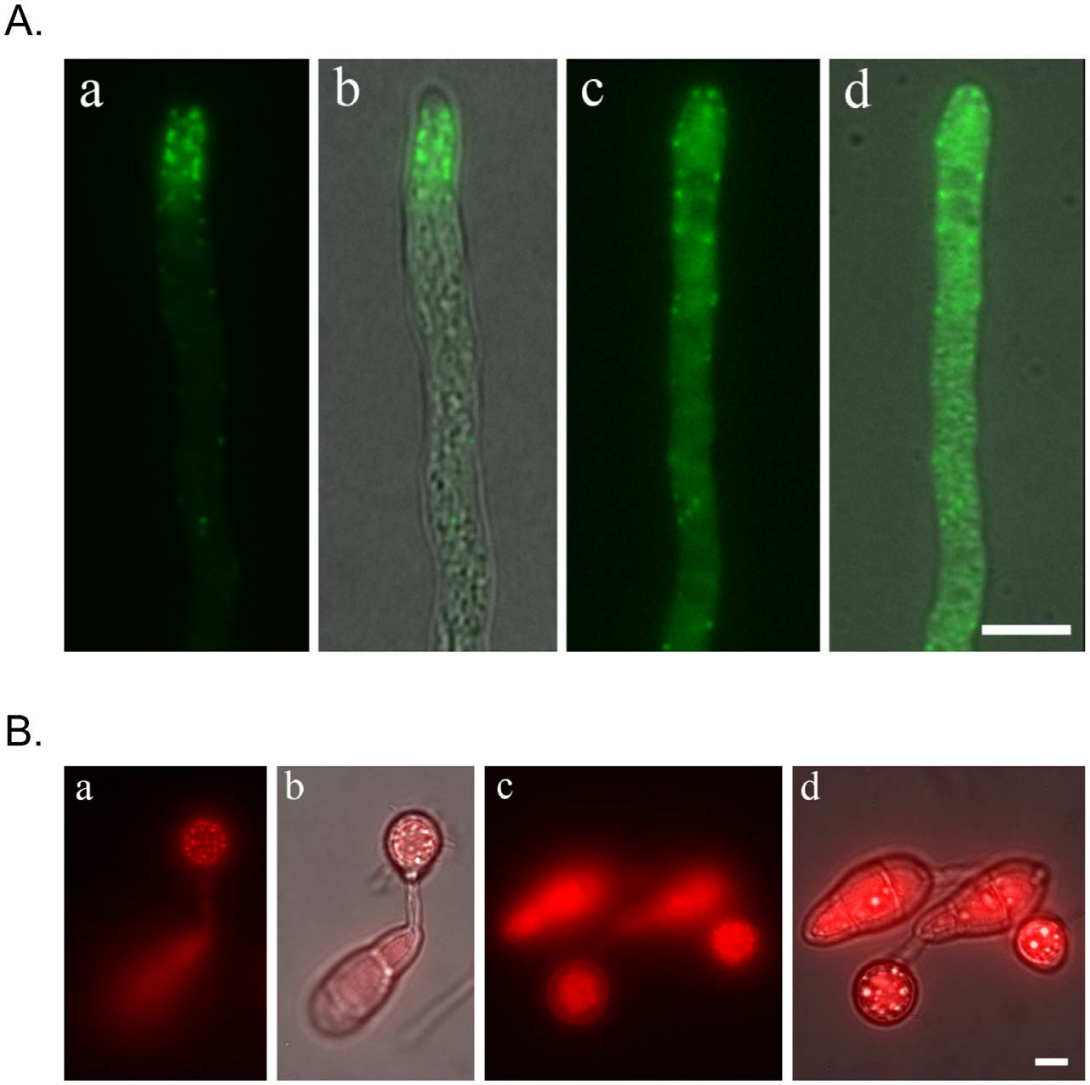
Cytochalasin A (CytA) treatment disrupted normal subcellular localization of Cap1 in vegetative hyphae (A) and during appressorium formation (B). **A.** Hyphae of the *CAP1*-GFP transformant treated with (**c** and **d**) or without (**a** and **b**) CytA were examined by DIC and fluorescence microscopy. Panels **a** and **c** were GFP images. Panels **b** and **d** were composites of GFP and DIC images. **B.** Conidia harvested from the *CAP1*-RFP transformant were incubated on hydrophobic surfaces for 16 h and examined by DIC and fluorescence microscopy. Panels **a** and **b** were non-treatment controls. Panels **c** and **d** were samples treated with CytA for 15 min. before examination. Panels **b** and **d** were composites of GFP and DIC images. Bar = 5 µm.

When the *CAP1*-GFP transformant XY61 was treated with cytochalasin A (CytA), an inhibitor of actin elongation, the subcellular localization pattern of Cap1-GFP in hyphae was completely disrupted. Instead of forming apical cortical patches, GFP signals were mainly observed in large cytoplasmic aggregates in the presence of CytA (Fig. 8A; Video S2, S3). CytA treatment also disrupted the normal localization pattern of Cap1 during appressorium formation (Fig. 8B; Video S4, S5), indicating that subcellular localization of Cap1 was changed from the actin-like pattern to the cytoplasm in both hyphae and appressoria.

We then examined the localization of Cap1-GFP in invasive hyphae. In rice leaf sheath cells, invasive hyphae formed by the *CAP1*-GFP contained bright spots of GFP signals in the cytoplasm (Fig. 7B). However, we failed to observe actin-like patches at the apical region of invasive hyphae. The difference between vegetative and invasive hyphae in the localization of Cap1-GFP may be related to differences in the role of actin cytoskeleton in hyphal growth. Bulbous invasive hyphae are morphologically distinct from vegetative hyphae and may lack the typical hyphal tip elongation mechanism.

### Localization of LifeAct-GFP proteins

To further confirm whether Cap1 has the actin-like distribution pattern, we generated a LifeAct-GFP construct and transformed it into the wild-type strain Guy11. In the resulting transformant LA31 (Table 1), LifeAct-GFP mainly localized to patches in the apical region of germ tubes and hyphal tips. It also displayed a localization pattern similar to Cap1-GFP in developing and mature appressoria (Fig. 7A). GFP signals were mainly observed in the periphery of developing appressoria and globular structures at the base of mature appressoria (Fig. 7A). In invasive hyphae formed by the transformant LA31 inside rice leaf sheath cells, we failed to observe actin-like patches or Cap1-like bright spots, further indicating that vegetative and invasive hyphae differ in the actin cytoskeleton organization at the tip.

**Figure 9:**
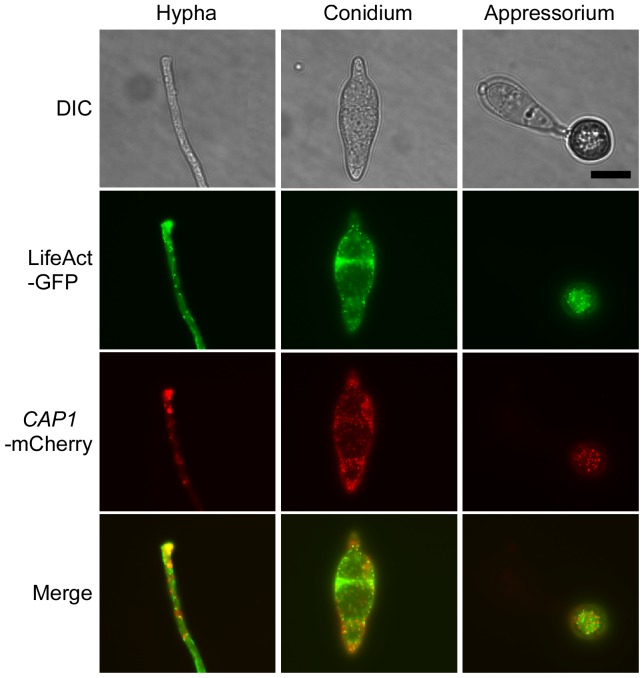
Assays for co-localization of LifeAct and Cap1. Vegetative hyphae, conidia, and appressoria (16 h) of transformant MC20 expressing the LifeAct-GFP and *CAP1*-mCherry constructs were examined under DIC and epifluorescence microscopy with GFP and mCherry-specific filters. The bottom panel was generated by merging the GFP and mCherry images.

While LifeAct binds to F-actin, the C-terminal domain of CAP proteins binds to G-actin . In this study, we observed that Cap1-GFP and LifeAct-GFP had a similar localization pattern. To distinguish their subcellular localization patterns, we generated the *CAP1*-mCherry construct and co-transformed it with LifeAct-GFP into Guy11. In the resulting transformant MC20 (Table 1) expressing both constructs, LifeAct-GFP and Cap1-mCherry had similar localization patterns, but they were not co-localized in most of the cytoplasmic regions in vegetative hyphae, conidia, and appressoria (Fig. 9).

### The ACB domain is essential for the function of *CAP1*

**Figure 10:**
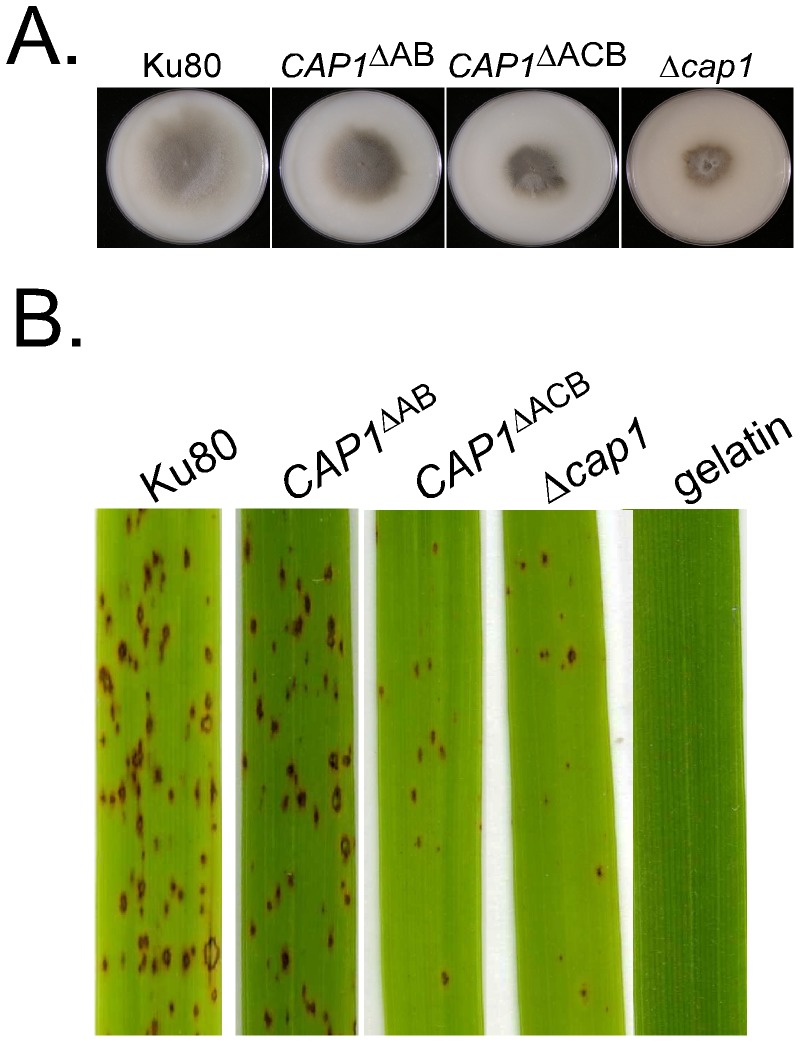
Functional analysis of the AB and ACB domains of *CAP1*. **A.** Growth and colony morphology of wild type Ku80, Δ*cap1*/*CAP1* ^ΔAB^ transformant, Δ*cap1*/*CAP1* ^ΔACB^ transformant, and Δ*cap1* mutant HC83. **B.** Rice leaves sprayed with conidia from Ku80, Δ*cap1*/*CAP1* ^ΔAB^ transformant, and Δ*cap1*/*CAP1* ^ΔACB^ transformant.

To determine its function, we generated the AB domain knock-in deletion construct in which residues 378–533 of *CAP1* were deleted. After transforming into the wild-type strain 70-15, the resulting *CAP1* ^ΔAB^ transformant HC10 (Table 1) was confirmed by Southern blot analysis (Fig. S4). On OTA plates, transformant HC10 grew faster than the *Δcap1* mutant but still slower than Ku80 (Fig. 10A). Conidiation was normal in the *CAP1* ^ΔAB^ transformant HC10 (Table 3), suggesting that the actin-binding domain is dispensable for the function of *CAP1* in conidiation. In infection assays with rice seedlings, transformant HC10 caused more lesions than the *Δcap1* mutant, but it was still reduced in virulence compared with Ku80 (Fig. 10B, Table 3). Similar results were observed in infection assays with barley seedlings. Therefore, although it is dispensable for conidiation, the AB domain of *CAP1* is important for the normal function of Cap1 and plays a role in plant infection and hyphal growth.

We also generated and transformed the *CAP1* ^ΔACB^ knock-in deletion construct into protoplasts of the wild-type strain Ku80. The phenotype of the *CAP1* ^ΔACB^ transformants XY111 and XY002 (Table 1) was similar to that of the *Δcap1* mutant. All the defects associated with deletion of *CAP1* were observed in the *CAP1* ^ΔACB^ transformants, including reduced appressorium formation, production of branching germ tubes (Fig. 10A), and reduced virulence (Fig. 10B). Thus, the ACB domain is essential for Cap1 function in *M. oryzae*.

### The P2 region, not the AB domain, is required for the subcellular localization of Cap1

**Figure 11:**
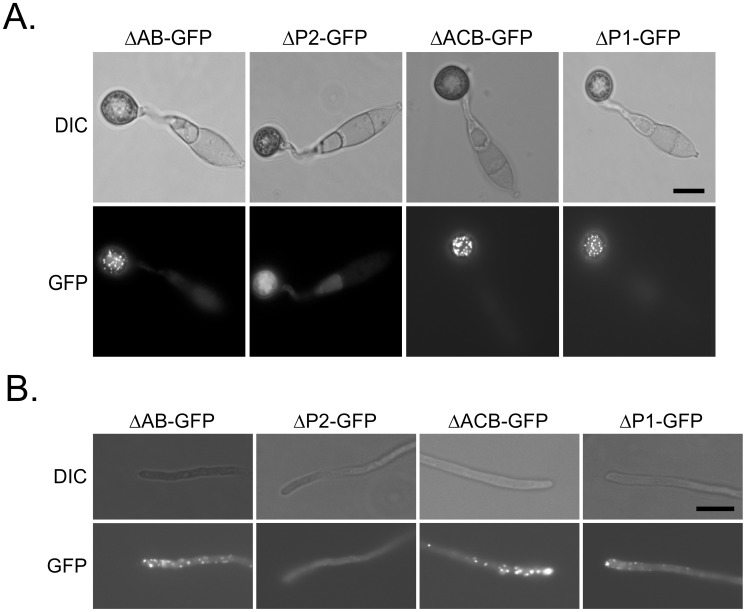
Functions of different domains in the localization of Cap1. Appressoria (16 h, **A**) and vegetative hyphae (**B**) of transformants of the Δ*cap1* mutant HC83 expressing the *CAP1* ^ΔAB^-, *CAP1* ^ΔP2^-, *CAP1* ^ΔAC^-, or *CAP1* ^ΔP1^-GFP construct were examined under DIC or epifluorescence microscopy. Bar = 10 µm.

We then generated the *CAP1* ^ΔAB^-GFP construct and transformed it into protoplasts of the *Δcap1* mutant. In the resulting C*AP1* ^ΔAB^-GFP transformant XY105 (Table 1), GFP signals had the Cap1^WT^-like localization pattern in appressoria (Fig. 11A) and hyphal tips (Fig. 11B). Therefore, the C-terminal AB domain is not essential for the subcellular localization of Cap1 in *M. oryzae*.

**Figure 12:**
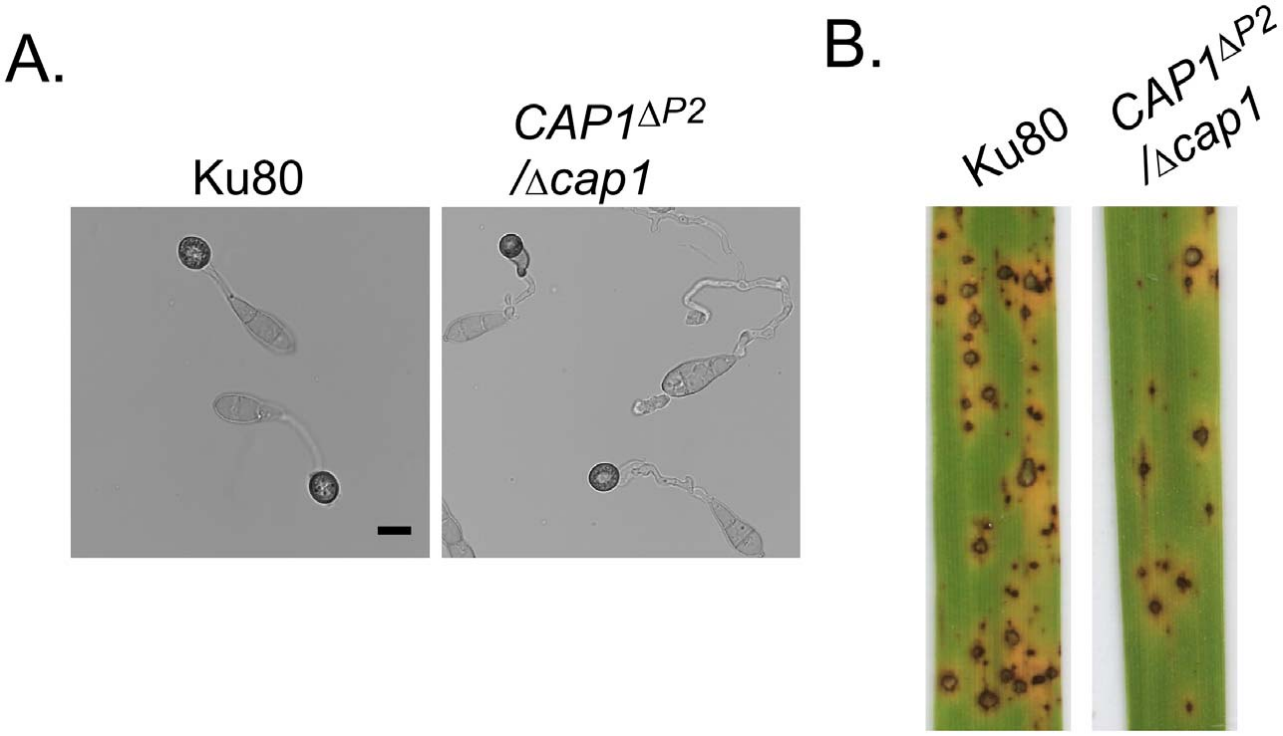
Functional analysis of P2 domain of *CAP1*. **A.** Appressorium formation assays with Ku80 and transformant XY244 (Δ*cap1*/*CAP1* ^ΔP2^) on hydrophobic surfaces by 24 h. **B.** Rice leaves sprayed with conidia of Ku80 and XY244. Transformant XY244 was significantly reduced in virulence.

Because the AB domain is dispensable for Cap1 localization, we transformed the *CAP1* ^ΔACB^-, *CAP1* ^ΔP1^-, and *CAP1* ^ΔP2^-GFP constructs that were deleted of residues 2–166, 257–290, and 355–377, respectively, into protoplasts of the *Δcap1* mutant. In transformants expressing the *CAP1* ^ΔACB^-GFP or *CAP1* ^ΔP1^-GFP constructs (Table 1), the localization of GFP signals was the same as the Cap1^WT^-GFP transformant in appressoria (Fig. 11A) and hyphal tips (Fig. 11B). In the *CAP1* ^ΔP2^-GFP transformants XY244 and XY001 (Table 1), GFP signals were observed in the cytoplasm in appressoria (Fig. 11A) and vegetative hyphae (Fig. 11B). The Cap1^WT^-like localization pattern of GFP-signals was not observed in conidia or invasive hyphae of the *CAP1* ^ΔP2^-GFP transformants (Fig. S5). These results indicate that the P2 region is responsible for the subcellular localization of Cap1 in *M. oryzae*. On hydrophobic surfaces, *CAP1* ^ΔP2^/*Δcap1* transformants produced long, branching germ tubes and were reduced in appressorium formation efficiency (Fig. 12A). In spray infection assays, the *CAP1* ^ΔP2^/*Δcap1* transformant XY244 formed 17.3±4.3 lesions/5 cm leave tip, which was significantly lower than 51.3±4.5 lesions/5 cm leave tip caused by the wild type (Fig. 12B). These results indicated that the P2 region of Cap1 is required for normal appressorium formation and virulence.

### Melanized compartments of conidia are induced by exogenous cAMP in the *CAP1* ^ΔAB^ transformant

**Figure 13:**
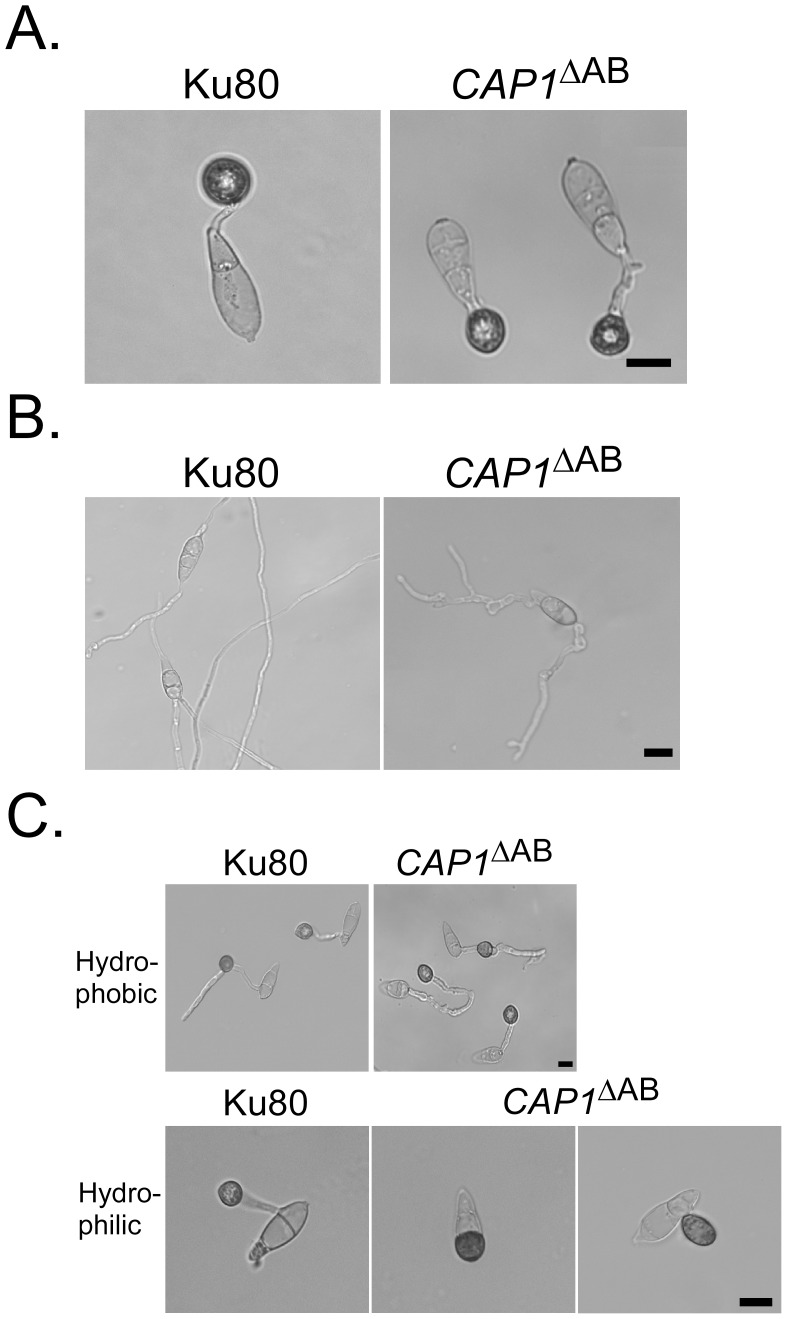
Appressorium formation assay with the *CAP1* ^ΔAB^ transformant. Conidia from Ku80 and the *CAP1* ^ΔAB^ transformant were incubated for 24 h on the hydrophobic (**A**) or hydrophilic (**B**) surface of GelBond membranes. **C.** The presence of 5 mM cAMP stimulated appressorium formation in the *CAP1* ^ΔAB^ transformant on hydrophobic and hydrophilic surfaces. Bar = 10 µm.

Because the AB domain of *CAP1* is important for full virulence, we assayed appressorium formation in the *CAP1* ^ΔAB^ transformant HC10 (Table 1). On hydrophobic surfaces, melanized appressoria were formed by transformant HC10 by 24 h (Fig. 13A). On hydrophilic surfaces, it produced branching germ tubes and subapical swollen bodies but not melanized appressoria (Fig. 13B). On hydrophilic surfaces, cAMP treatment induced appressorium formation on short germ tubes in the *CAP1* ^ΔAB^ strain (Fig. 13C). Some of the melanized appressoria were formed immediately adjacent to the germinating conidia without visible germ tubes (Fig. 13C). Interestingly, approximately 40% of the *CAP1* ^ΔAB^ conidia became melanized in one of the conidium compartments in the presence of 5 mM cAMP (Fig. 13C). In hundreds of conidia examined, only one conidium compartment was melanized. The other two compartments in the same conidium often were empty and dead by 24 h, which is similar to what has been observed in conidia after appressorium formation [40]. However, exogenous cAMP failed to induce the formation of melanized conidium compartments in the *CAP1* ^ΔAB^ transformant on hydrophobic surfaces (Fig. 13C), indicating that the surface recognition signal could suppress the effect of cAMP treatment on improper melanization of conidium compartments.

### *PMK1* is required for the melanization of conidium compartments induced by cAMP

**Figure 14:**
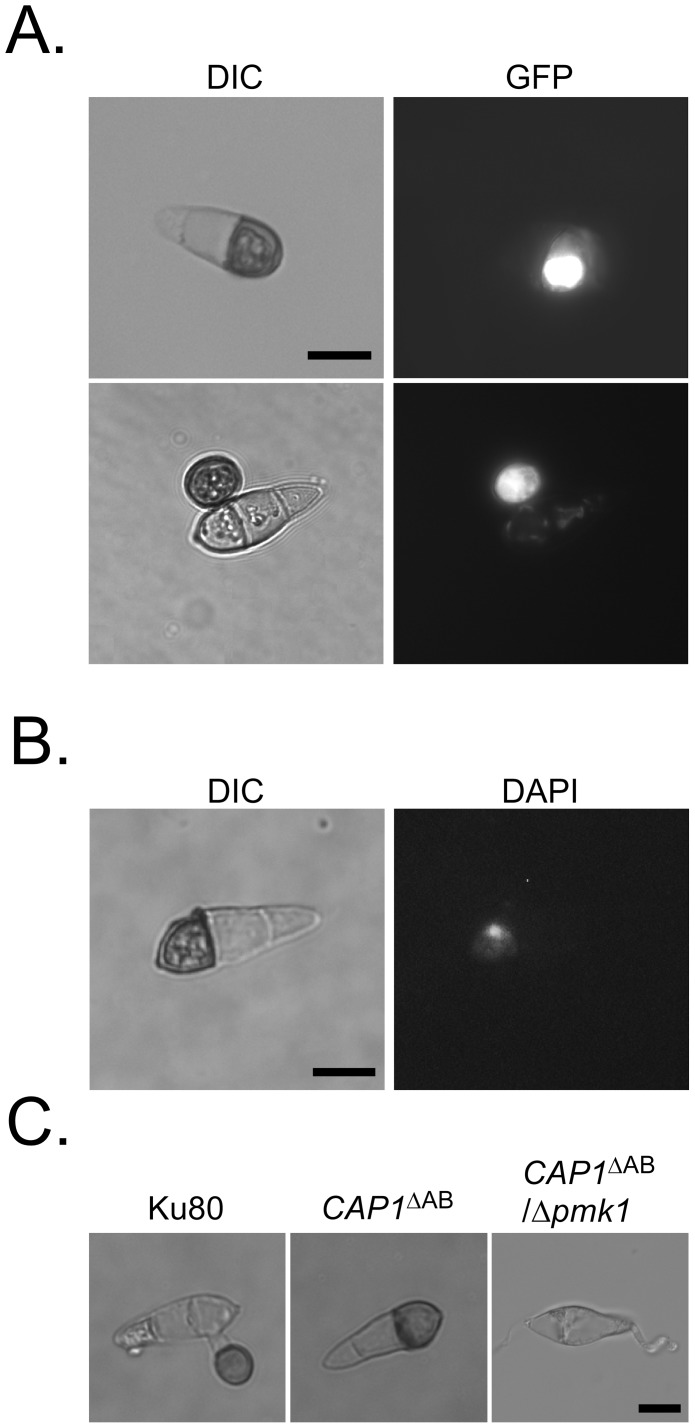
Melanized conidium compartments were appressorium-like structures. **A.** Conidia of a transformant of the Δ*cap1*/*CAP1* ^ΔAB^ mutant expressing the *GAS2*-GFP fusion construct were incubated on the hydrophilic surface of GelBond membranes in the presence of 5 mM cAMP. GFP signals were observed in the melanized conidium compartments and appressoria at 24 h. **B.** DAPI staining of melanized conidium compartments formed by the Δ*cap1*/*CAP1* ^ΔAB^ transformant. **C.** Conidia from the Ku80, Δ*cap1*/*CAP1* ^ΔAB^, and Δ*pmk1*/*CAP1* ^ΔAB^ strains HC1-39 were assayed for appressorium formation in the presence of 5 mM cAMP on hydrophilic surfaces. Bar = 10 µm.

To determine whether melanized conidium compartments were similar to appressoria, we introduced the *GAS2*-GFP construct [41] into the *CAP1* ^ΔAB^ transformant. *GAS2* encodes a cytoplasmic protein specifically expressed during late stages of appressorium formation (Fig. S6A) [41]. The resulting transformant CGS (Table 1) expressed GFP signals in the melanized conidium compartments, suggesting that those were appressorium-like structures (Fig. 14A). When stained with DAPI, melanized conidium compartments induced by 5 mM cAMP contained a single nucleus (Fig. 14B). The other two compartments were devoid of DAPI staining at 24 h. These results indicate that exogenous cAMP induced the formation of appressorium-like structures in conidia without germination.

We also transformed the *CAP1* ^ΔAB^ construct into the *Δpmk1* mutant nn78 that was defective in appressorium formation on hydrophobic surfaces [37]. In the resulting *Δpmk1*/*CAP1* ^ΔAB^ transformant HC1-39, cAMP treatment failed to induce the formation of melanized conidium compartments (Fig. 14C). Exogenous cAMP also failed to induce appressorium formation in the *Δpmk1*/*CAP1* ^ΔAB^ transformant on hydrophobic surfaces (Fig. S6B). Therefore, like appressorium formation, the development of melanized conidium compartments is under the control of Pmk1.

## Discussion

The cAMP-PKA pathway is involved in surface recognition, appressorium turgor generation, and invasive growth in *M. oryzae* [8], [15], [37]. In other plant pathogenic fungi, cAMP signaling also has been implicated in the regulation of various differentiation and infection processes [42], [43]. In this study, proteins associated with the Mac1 adenylate cyclase in *M. oryzae* were identified by affinity purification. One of the Mac1-interacting proteins was the adenylyl cyclase associated protein Cap1. CAP proteins are well conserved from yeast to humans [44]. The Cap1 protein of *M. oryzae* has the typical structural features (Fig. 1A) of CAPs. The well-conserved N- and C-terminal regions of *CAP1* are rich in α-helix and β-sheet structures, which are known to interact with adenyly cyclase and actin monomers, respectively [45], [46]. Recently, the N-terminal region of yeast and mammalian CAP proteins were reported to bind to the cofilin-actin complex, which promotes the actin turnover [19], [47]. We also noticed that the C-terminal region of Mac1 has the heptad motif that is sufficient for coiled-coiled interactions with Cap1 (Fig. 1A; Fig. S1B). Unlike their association with actin cytoskeleton, the function of CAP proteins in regulating the adenylyl cyclase activity was not conserved in animals and plants [10], [44], [48].

Adenylyl cyclase was reported to be an integral transmembrane protein in mammalian cells [32] and a peripheral membrane protein in the budding yeast [33]. In BiFC assays, Cap1 and Mac1 appeared to weakly interact with each other in vegetative hyphae, conidia, and appressoria. Their interaction was enhanced during appressorium formation and localization of YFP signals to the cytoplasmic membrane was observed (Fig. 2). In *S. cerevisiae*, Cyr1 adenylate cyclase is activated by Ras2 and enriched in a subset of G-protein-containing fractions of cytoplasm membrane [49]. Although it is not essential, the interaction of Srv2 with Cyr1 and posttranslationally modified Ras2 [50] is important for the Ras-dependent activation of Cyr1. Ras proteins are known for their membrane-anchoring functions in recruiting effector molecules to the cytoplasma membrane [51]. In yeast, disruption of the *IRA1* Ras GTPase gene released the majority of adenylate cyclase activities (90%) to the cytosol, which was significantly more than 20% in the wild-type cells [52]. In *M. oryzae*, the interaction of Cap1 with Ras2 and Mac1 may also play a role in the recruitment of Mac1 to the cytoplasm membrane and its activation.

The *Δcap1* mutant had a reduced intracellular cAMP level, indicating that *CAP1* is involved in the cAMP-PKA pathway, possibly by regulating the activity of adenylate cyclase. Cap1 interacted with Mac1 in yeast two-hybrid and co-IP assays (Fig. 1B, 1C). In *S. cerevisiae*, a small N-terminal region of Srv2 is sufficient for its association with Cyr1 and function in the Ras-adenylyl cyclase pathway [31]. L16P and R19T mutations of Srv2 resulted in attenuated cAMP signaling and reduced cortical actin patch localization [16]. Our data showed that the N-terminal region of *CAP1* is essential for its interaction with Mac1 but not its actin-like localization pattern in *M. oryzae* (Fig. S2). The phenotype of the *CAP1* ^ΔACB^/Δ*cap1* transformant was similar to that of the Δ*cap1* mutant, further confirming the importance of the ACB domain in Cap1 function. Because the Δ*cap1* mutant had a higher intracellular cAMP level than that of the *mac1* mutant and it still recognized hydrophobic surfaces for appressorium formation, we conclude that *CAP1* is not essential for surface recognition. The full activation of Mac1 was reduced but not completely blocked in the *cap1* mutant. In *S. cerevisiae*, Srv2 plays an important role in normal activation of Cyr1 and the direct interaction between Cyr1 and Ras2. Srv2 was first identified as a suppressor of a hyper-activated *RAS2* ^V19^ allele in yeast, indicating their genetic association [29]. We also found that deletion of *CAP1* suppressed the improper formation of melanized appressoria on hydrophilic surfaces in the *RAS2* ^DA^ transformant (Fig. 4). Therefore, Cap1 may also facilitate the interaction of Ras2 with Mac1 in *M. oryzae*.

On hydrophobic surfaces, most of the germ tubes of the Δ*cap1* mutant were morphologically abnormal (Fig. 3A). It appeared that these germ tubes attempted to form appressoria but failed to be arrested in tip growth. In addition, branching germ tubes were often observed in the mutant. Because exogenous cAMP suppressed these defects, maintaining the normal intracellular cAMP level must be important for the regulation of germ tube growth and branching (Fig. 5C). It is likely that Cap1 plays a critical role in the proper activation of Mac1 and regulation of normal germ tube growth and appressorium formation. The Δ*cap1* mutant may be defective in actin cytoskeleton reorganization in response to surface recognition signals and maintaining polarized tip growth in germ tubes. However, the *CAP1* ^ΔAB^ transformant produced relatively normal germ tubes and appressoria (Fig. 13). It did not form branching germ tubes, suggesting that the AB domain is dispensable for the suppression of germ tube branching after the initiation of appressorium formation. Deletion of the AB domain had some minor effects on but did not eliminate the cortical patch localization of Cap1 (Fig. 11).

Domain deletion analysis indicated that the P2 region but not the AB domain is responsible for the subcellular localization of Cap1 in *M. oryzae*. Furthermore, the P2 deletion mutant produced branching germ tubes on hydrophobic surfaces and had a reduced virulence, indicating that loss of the actin-like localization pattern resulted in phenotypes similar to those of the *CAP1* ^ΔACB^ transformant although to a less degree. In *S. cerevisiae* and *C. albicans*, the AB domain of CAPs also is dispensable for its association with actin cortical patches and cytoskeletal organization [24], [53]. The P2 region likely binds to the SH3 domain of Abp1, which may facilitate the localization of Cap proteins to sites of actin rearrangement. In yeast, the P2 motif is important for the binding of Srv2 with G-actin and directing its localization to cortical actin patches [47], [54]. The P1 region of Srv2 may be responsible for profiling-binding but dispensable for actin-binding [16], [19]. Consistent with these reports, we found that the P1 region was dispensable for the actin-like localization pattern of Cap1 in *M. oryzae*.

In human pathogens, the *cap1/cap1* mutant of *C. albicans* and the Δ*aca1* mutant of *C. neoformans* are non-pathogenic [23], [24]. The Δ*cap1* mutant of *M. oryzae* was significantly reduced in virulence but still incited few small lesions on rice and barley leaves. In the rice blast fungus, *MAC1* is essential for virulence. It is likely that reduced virulence of the Δ*cap1* mutant is directly related to the function of *CAP1* in the cAMP-PKA pathway. Lesions caused by the Δ*cap1* mutant rarely had extensive necrosis indicating its defects in invasive growth in plant cells (Fig. 6B). *M. oryzae* is a hemibiotrophic pathogen that has been used as a model for studying fungal–plant interactions [55]. However, it is not clear when and how the transition from the biotrophic phase to necrotrophic growth occurs. *CAP1* may play a role in regulating the biotrophic-necrotrophic transition. It is also possible that *CAP1* plays a role in maintaining normal invasive growth. Bulbous invasive hyphae of *M. oryzae* are morphologically distinct from vegetative hyphae and may involve different hyphal growth mechanisms.

In yeast, Srv2 functions as an adaptor protein for the translocation of adenylate cyclase to actin cortex patches but this translocation is not essential for the cAMP signaling pathway [16]. In higher eukaryotes, the localization of CAP is species specific. In *Dictyostelium discoideum*, CAP localizes near the plasma membrane in resting cells but is remobilized during cell movement [56]. In mammalian cells, h*CAP1* is distributed throughout cytoplasm but concentrated at actin-rich membrane ruffles and lamellipodia of migrating fibroblasts [20], [57]. In cells induced for apoptosis, Cap1 was rapidly translocated to the mitochondrium independent of caspase activation [58]. In this study, we found that Cap1 in *M. oryzae* also had an actin-like localization pattern in hyphae and germ tubes. Cap1 mainly localized to patches in the apical region of vegetative hyphae or germ tubes and the base of appressoria surrounding the appressorium pore area. LifeAct had similar localization patterns with Cap1-GFP, but they usually were not co-localized to the same cytoplasmic regions, which is consistent with their differences in binding to different forms of actins [27], [47]. Cap1 may also function as an adaptor protein for translocating Mac1 to the actin cortex in *M. oryzae*. Interestingly, localization of Cap1 or LifeAct to the apical region of invasive hyphae was not observed in the same transformants that displayed actin-like patterns in vegetative hyphae. The role of the actin cytoskeleton in hyphal tip growth may differ between vegetative and invasive hyphae.

Expression of the *CAP1* ^ΔAB^ allele partially suppressed the Δ*cap1* deletion mutant in germ tube growth and virulence. Interestingly, the *CAP1* ^ΔAB^ transformant formed melanized conidial compartments when treated with 5 mM cAMP on hydrophilic surfaces. Because *GAS2* [41] was specifically expressed in appressoria, those melanized compartments appeared to be appressorium-like structures. In *M. oryzae*, mitosis is known to be a prerequisite for appressorium development [59], and DNA replication is necessary for the initiation of appressorium formation [40], [60]. In the *CAP1* ^ΔAB^ transformant, only one nucleus was observed in the melanized compartments, suggesting that mitosis may not occur before these compartments become melanized in responses to exogenous cAMP. In *S. cerevisiae*, activation of the cAMP-dependent pathway causes cells to undergo unipolar growth, a process coupling with elongated growth that is controlled by the filamentous MAPK pathway [61]. It is likely that *PMK1* was over-activated by exogenous cAMP when the AB domain of *CAP1* was deleted in *M. oryzae*. Deletion of the AB domain of *CAP1* in the *pmk*1 mutant failed to cause the formation of melanized conidium compartments, indicating that the formation of melanized conidium compartments requires the presence of functional Pmk1 (Fig. 13C).

**Figure 15:**
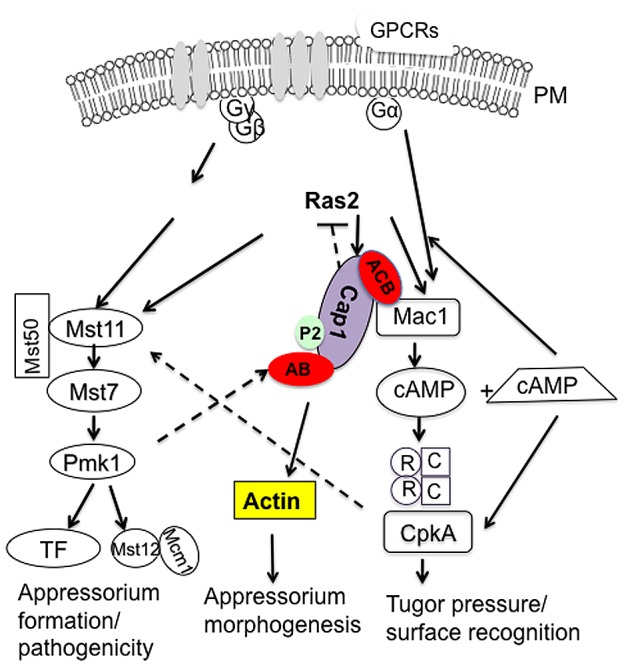
A hypothetical model of the function of Cap1. In *M. oryzae*, cAMP signaling is the secondary messenger for surface sensing to initiate appressorium formation. Although their exact relationship is not clear, surface sensing signals must be transduced from the cAMP signaling to the Pmk1 MAP kinase pathway, which regulates appressorium development and plant penetration. Ras2 functions upstream both Pmk1 and cAMP signaling. Cap1 directly interacts with Mac1 and plays a role in the activation of Mac1, which may function downstream of Ras or MagB. In cells lacking Cap1, Mac1 cannot be fully activated, which leads to the reduced intracellular cAMP level and reduced appressorium formation. Exogenous cAMP partially suppresses the phenotype of *Δcap1* mutant. Reduced intracellular cAMP may somehow affect the proper activation of Pmk1, which can explain the formation of subapical swollen bodies in the *Δcap1* mutant. Cap1 may also interact with actin and is involved in cytoskeleton reorganization during appressorium morphogenesis. In addition, it is likely that Cap1 is involved in the feedback inhibition of Mac1 and Ras2 signaling by the activated Pmk1. Deletion of *CAP1* may affect this process and result in the formation of branched germ tubes. The AB domain may play a critical role in the feedback inhibition by directly interact with Pmk1 or by being phosphorylated by Pmk1. Meanwhile, the Cap1^ΔAB^ protein may be hyperactive in activating Mac1 via Ras2. Therefore, exogenous cAMP may overstimulate Ras signaling in the *CAP1* ^ΔAB^-GFP transformant and result in the inappropriate activation of the Pmk1 pathway, which may be responsible for the formation of melanized conidium compartments and appressoria in the absence of visible germ tubes.

Melanized conidium compartments were not observed in the Δ*cap1* mutant when treated with 5 mM cAMP. When the entire *CAP1* gene was deleted, exogenous cAMP stimulated appressorium formation on germ tubes. Therefore, deletion of the AB domain or the entire *CAP1* gene had different effects on responses to cAMP treatment. One possible explanation for this puzzling observation is that, in addition to being involved in the activation of Mac1 and Ras2 for appressorium formation, Cap1 is involved in the feedback inhibition or down-regulation of Ras2 signaling when Pmk1 is activated (Fig. 15). Ras2 likely functions upstream from both the cAMP signaling and Pmk1 MAPK pathway in *M. oryzae*. The Cap1^ΔAB^ protein may be defective in the feedback inhibition of Mac1 and Ras2, but retains the ability to regulate appressorium morphogenesis in response to exogenous cAMP. Therefore, cAMP treatment may overstimulate Ras signaling in the *CAP1* ^ΔAB^ transformant and result in the inappropriate activation of the Pmk1 pathway, which may be responsible for the formation of melanized conidium compartments and appressoria without visible germ tubes (Fig. 15). Interestingly, serine residue 450 of Cap1 was predicted to be a putative MAPK phosphorylation site (over 50% probability) by Kinasphos2.0. This serine residue is conserved in its orthologs from *S. cerevisiae*, *C. albicans*, mammalian cells, and other few filamentous fungi examined. It is possible that Pmk1, when activated, phosphorylates or interacts with Cap1 at the AB domain, which is involved in the down-regulation of Ras signaling. Therefore, further characterization of the functional relationships among Ras2, Mac1, and Cap1 may provide necessary information to better understand the interactions between the cAMP and *PMK1* signaling pathways during appressorium morphogenesis and plant infection.

## Materials and Methods

### Strains and culture conditions

The wild type and mutant strains of *M. oryzae* (Table 1) were cultured on oatmeal agar (OTA) plates at 25°C under fluorescent light for conidiation and preserved on filter paper at −20°C as described [37]. For transformation selection, hygromycin (Calbiochem, La Jolla, CA) and zeocin (Invitrogen, Carlsbad CA) were added to final concentrations of 250 µg/ml and 200 µg/ml, respectively. Monoconidial culture isolation, measurement of growth rate and conidiation were performed as previously described [14], [62]. Vegetative hyphae from 3-day-old 5×YEG (0.5% yeast, 1% glucose) cultures were used for DNA, RNA, and protein isolation. Intracellular cAMP was assayed as described [12], [63] with the cAMP enzyme immunoassay system (Amersham Pharmacia Biotech, Piscataway, NJ).

### Construction of the *CAP1* gene replacement vector and domain deletion alleles

The upstream and downstream flanking sequences of *CAP1* were amplified with primer pairs 1F/2R and 3F/4R (Table S1), respectively. The resulting PCR products were digested and ligated with the hygromycin-phosphotransferase (*hph*) cassette released from pCX63 as described [36]. The final gene replacement construct was amplified with primers 1F and 4R and directly transformed into protoplasts of Ku80 [64]. The putative *Δcap1* mutants were identified by PCR and confirmed by DNA blot analysis. For complementation assays, the full-length *CAP1* gene amplified with primers CF and CR was cloned between the *Not*I and *Xho*I sites of pYK11 [36] as pXY63.

The same ligation PCR approach [36] was used to generate the *CAP1* ^ΔAB^ construct and transformants. The upstream and downstream flanking sequences were amplified with primer pairs AB1F/AB2R and 3F/4R, respectively. After ligation with the *hph* gene, the ligation PCR product was transformed into Ku80. The *CAP1* ^ΔAB^ transformants were confirmed by Southern blot analysis to be deleted of the actin-binding domain (378–533 aa).

### Appressorium formation, penetration, and plant infection assays

Conidia were harvested from 10-day-old OTA cultures and resuspended to 5×10^4^ conidia/ml in H_2_O. For appressorium formation assays, 50-µl droplets of conidial suspensions were placed on glass cover slips (Fisher Scientific, St. Louis, IL) or GelBond membranes (Cambrex, East Rutherford, NJ) and incubated at 25°C. To assay its stimulatory effect on appressorium formation, cAMP was added to the final concentration of 5 mM to conidium suspensions. Penetration assays with onion epidermis and rice leaf sheaths were performed as described [8], [65], [66]. The growth of invasive hyphae was examined 48–72 h post-inoculation (hpi).

For infection assays, conidia were resuspended to 5×10^4^ conidia/ml in 0.25% gelatin. Two-week-old seedlings of CO-39 were used for spray or injection infection assays as described [62], [67]. Lesion formation was examined 7-day post-inoculation (dpi).

### Complementation of the yeast *srv2* mutant

The *S. cerevisiae srv2* deletion mutant was derived from *MATα* strain BY4741 (Open Biosystems, Huntsville, AL). The entire *CAP1* open reading frame (ORF) was amplified from 1st strand cDNA of strain 70-15 and cloned into pYES2 (Invitrogen). The resulting construct, pYES2-*CAP1*, was transformed into the *srv2* mutant with the alkali-cation yeast transformation kit (MP Biomedicals, Solon, OH). Ura3^+^ transformants were isolated and assayed for invasive growth and sensitivity to 5 mM H_2_O_2_ or 1 M NaCl as described [35].

### Generation of GFP or 3×FLAG fusion constructs and *CAP1* domain deletion alleles

The entire *CAP1* gene was amplified and cloned into the pHZ126 and pDL2 [4], [68] vectors by the yeast gap repair approach [69]. Similar approaches were used to generate the *CAP1* ^ΔACB^, *CAP1* ^ΔAB^, *CAP1* ^ΔP1^, and *CAP1* ^ΔP2^ alleles that were deleted of amino acid residues 2–166, 378–534, 257–290, and 355–377, respectively. All of the primers used in the construction of these mutant alleles are listed in Table S1. All of the resulting *CAP1*-3×FLAG (pXY60), *CAP1*-GFP (pXY61), *CAP1* ^ΔACB^-3×FLAG (pXY109), *CAP1* ^ΔACB^-GFP (pXY94), *CAP1* ^ΔAB^-3×FLAG (pXY110), *CAP1* ^ΔAB^-GFP (pXY105), *CAP1* ^ΔP1^-GFP (pXY95), and *CAP1* ^ΔP2^-GFP (pXY244) fusion constructs were confirmed by sequencing analysis and transformed into the Δ*cap1* mutant HC83.

### Co-immunoprecipitation and affinity purification

To confirm the interaction between *MAC1* and *CAP1 in vivo*, the C-terminal region of *MAC1* (1898–2016 aa, Mac1^CT^) was cloned into pHZ126 by the yeast gap repair approach [69]. The resulting construct, pXY165, was co-transformed with pXY61 into protoplasts of strain 70-15. It also was co-transformed with pXY94 (*CAP1* ^ΔACB^-GFP) into 70-15 to detect the Mac1-Cap1 interaction. Total proteins were isolated from transformants expressing both Mac1^CT^-3×FLAG and Cap1-GFP/Cap1^ΔACB^-GFP and incubated with anti-Flag M2 affinity resins (Sigma). Proteins bound to M2 resins were eluted after a series of washing steps as described [67], [70]. Western blots of total proteins and elution from the M2 resins were detected with anti-FLAG (Sigma-Aldrich) and anti-GFP (Roche) antibodies using the ECL Supersignal system (Pierce, Rochford, IL).

For affinity purification, total proteins were isolated from transformants expressing the *CAP1*-3×FLAG, *CAP1* ^ΔACB^-3×FLAG, and *CAP1* ^ΔAB^-3×FLAG constructs and incubated with anti-FLAG M2 affinity resins. After washing three times with 1×TBS (pH 7.4) buffer, three times with 50 mM TMAB, and three times with ddH_2_O, proteins were eluted with 0.1% Rapigest as described [28]. The elution proteins were digested with trypsin [71] and analyzed by Nanoflow liquid chromatography tandem mass spectrometry (nLC-MS/MS) as described [70]. The resulting MS/MS data were used to search against the non-redundant *M. oryzae* protein database at NCBI.

### Construction of the LifeAct-GFP and *CAP1*-mCherry transformants

The first 17 amino acid residues (MGVADLIKKFESISKEE) of Abp140 of *S. cerevisiae*, named LifeAct, is an F-actin marker for higher eukaryotes [27]. The LifeAct sequence was amplified with primers LifeActF and LifeActR (Table S1) and cloned into pDL2 and pFL1 by yeast gap repair [69]. The resulting LifeAct-Gly (10)-GFP constructs pXY198 (HygR) were confirmed by sequencing analysis and transformed into Guy11. The *CAP1*-mCherry construct pXY210 was generated by cloning the *CAP1* fragment into pXY201, which was a vector generated in this study by replacing the GFP sequence on pYP1 [68] with the mCherry sequence.

### Yeast two-hybrid assays

The bait construct of *CAP1* was generated by cloning full-length *CAP1* ORF amplified with primers *CAP1*-YF and *CAP1*-YR (Table S1) into pAD-GAL4 (Stratagene, La Jolla, CA). The C-terminal region of *MAC1* (1926–2160 aa) was amplified with primers MAC1-YF and MAC1-YR and cloned into pBD-GAL4-2.1 as the prey construct. The resulting prey and bait constructs were transformed in pairs into yeast strain YRG-2 (Stratagene). The Trp^+^ and Leu^+^ transformants were isolated and assayed for growth on SD-Trp-Leu-His medium and the expression of LacZ reporter gene was detected according to the instruction provided by Stratagene. Yeast transformants expressing the *MST11*-bait and *MST50*-prey, *PMK1*-bait and *MST50*-prey constructs [12], [14] were used as the positive and negative controls, respectively.

### BiFC assays for the Cap1-Mac1^CT^ interaction

The *CAP1*-CYFP fusion construct pXY178 was generated by cloning the *CAP1* fragment amplified with primers FlagF and CBFR into pHZ65 [13]. The C-terminal region of *MAC1* was amplified with primers Mac1-FLF and MBR and cloned into pHZ68 [13] to generate the *MAC1*-NYFP fusion construct pXY179. Plasmids pXY178 and pXY179 were co-transformed into protoplasts of 70-15. Transformants resistant to both hygromycin and zeocin were isolated and confirmed by PCR and Southern blot analyses. YFP signals were examined with a Nikon 800 epifluoresence microscope.

### GenBank accession numbers

Sequence data for genes described in this article can be found in the GenBank under the following accession numbers: *M. oryzae CAP1* (XM_363796.2), *MAC1* (XM_365053.1), *S. cerevisiae SRV2* (AAA35094.1), *Homo sapiens* CAP1 (CAG33690), *C. albicans CAP1* (AAD42978.1)

## Supporting Information

Figure S1 The MAC1-3×FLAG transformant and sequence alignment of CAPs from selected fungi. **A.** Western blots of total proteins and proteins eluted from anti-FLAG M2 beads from transformant MCF12 expressing the *MAC1*-3×FLAG construct were detected with an anti-FLAG antibody. **B.** The amino acid alignment of N-terminal CAP and C-terminal adenylate cyclase (AC). Mo: *Magnaporthe oryzae*; Fg: *Fusarium graminearum*; Nc: *Neurospora crassa*; An: *Aspergillus nidulans*; Sp: *Schizosaccharomyces pombe*; Sc: *Saccharomyces cerevisiae*; Ca: *Candida albicans*.
(TIF)

Figure S2 Co-immunoprecipitation assays. Western blots of total proteins isolated from transformant DCN22 (expressing the *CAP1^−^* ^ΔACB^-GFP and *MAC1* ^CT^-3×FLAG constructs) and proteins eluted from anti-FLAG M2 beads were detected with an anti-FLAG or anti-GFP antibodies. ACB: Putative N-terminus AC-binding domain of Cap1.
(TIF)

Figure S3 Generation of the *CAP1* gene replacement construct and mutant. **A.** The genomic region of the *CAP1* gene and PCR fragments used for constructing the gene replacement mutants and hybridization. PCR primers are marked with small arrows. N, *Nco*I. **B.** Blots of *Nco*I-digested genomic DNA of Ku80 and the Δ*cap1*mutant were hybridized with probe 1 and probe 2 (see Fig. S3A). Probe 1 detected a 6.2-kb band in Ku80 but a 3.7-kb band in the Δ*cap1* mutant. When hybridized with probe 2, only strain Ku80 had the 6.2-kb band. **C.** One-week-old CM cultures of Ku80, Δ*cap1* mutant, and Δ*cap1/CAP1* transformant.
(TIF)

Figure S4 Generation of the *CAP1* ^ΔAB^ mutant. **A.** Small arrows mark the PCR primers used to amplify genomic fragments for generating the *CAP1* ^ΔAB^ allele and probes for hybridization. N, *Nco*I. **B.** Blots of *Nco*I-digested genomic DNA of Ku80 and *CAP1* ^ΔAB^ mutants were hybridized with probe 1 and probe 2. Probe 1 detected a 6.2-kb band in Ku80 but a 5.0-kb band in the *CAP1* ^ΔAB^ mutant. When hybridized with probe 2, only strain Ku80 had the 6.2-kb band.
(TIF)

Figure S5 Localization of Cap1^ΔP2^-GFP in germ tubes, young appressoria, and invasive hyphae. **a–b**) Germ tubes and appressoria of the *CAP1* ^ΔP2^-GFP transformant examined by DIC and epifluoresence microscopy. **c–d**) Invasive hyphae of the *CAP1* ^ΔP2^-GFP transformant formed inside onion epidermal cells. ap: appressorium; cw: onion cell wall. Bar = 10 µm.
(TIF)

Figure S6 **A.** Localization of *GAS2*-GFP in appressoria formed on hydrophobic (upper panel) and hydrophilic surfaces in the presence of 5 mM cAMP. **B.** Appressorial formation assays with the *CAP1* ^ΔAB^/Δ*pmk1* transformant on hydrophobic surfaces with or without exogenous cAMP.
(TIF)

Table S1 PCR primers used in this study.
(DOCX)

Video S1 GFP signals in an appressorium (from top to bottom) formed by the *CAP1*-GFP transformant on the hydrophobic surface. Bar = 5 µm.
(MPG)

Video S2 Dynamic movement of Cap1-GFP in hyphae with CytA treatment. Time, HH:MM:SS. Bar = 5 µm.
(AVI)

Video S3 Dynamic movement of Cap1-GFP in hyphae without CytA treatment. Time, HH:MM:SS. Bar = 5 µm.
(AVI)

Video S4 The localization of Cap1-mCherry during appressorium formation with CytA treatment. The appressoria, after inoculated on hydrophobic surfaces for 16 h were treated with CytA. Time, HH:MM:SS. Bar = 5 µm.
(AVI)

Video S5 The localization of Cap1-mCherry during appressorium formation without CytA treatment. Time, HH:MM:SS. Bar = 5 µm.
(AVI)
