# Supplementary figures and images for: The Cyclase-Associated Protein Cap1 Is Important for Proper Regulation of Infection-Related Morphogenesis in Magnaporthe oryzae
Source: PLoS Pathog. 2012 Sep 6;8(9):e1002911. doi: 10.1371/journal.ppat.1002911 (PMC3435248; doi:10.1371/journal.ppat.1002911)

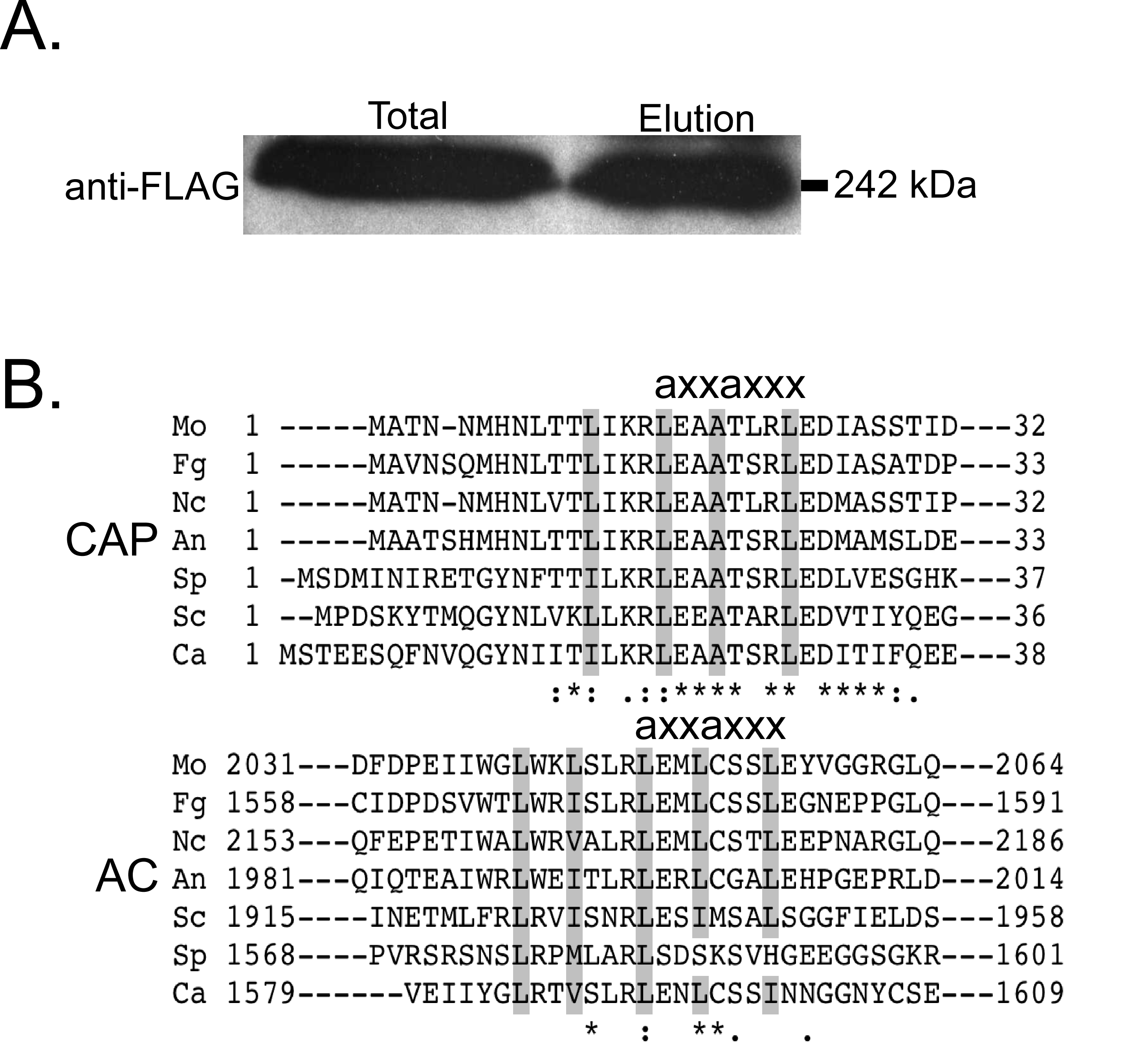

Supplement: Figure S1 — The MAC1-3×FLAG transformant and sequence alignment of CAPs from selected fungi. A. Western blots of total proteins and proteins eluted from anti-FLAG M2 beads from transformant MCF12 expressing the MAC1-3×FLAG construct were detected with an anti-FLAG antibody. B. The amino acid alignment of N-terminal CAP and C-terminal adenylate cyclase (AC). Mo: Magnaporthe oryzae; Fg: Fusarium graminearum; Nc: Neurospora crassa; An: Aspergillus nidulans; Sp: Schizosaccharomyces pombe; Sc: Saccharomyces cerevisiae; Ca: Candida albicans. (TIF) [file ppat.1002911.s001.tif]

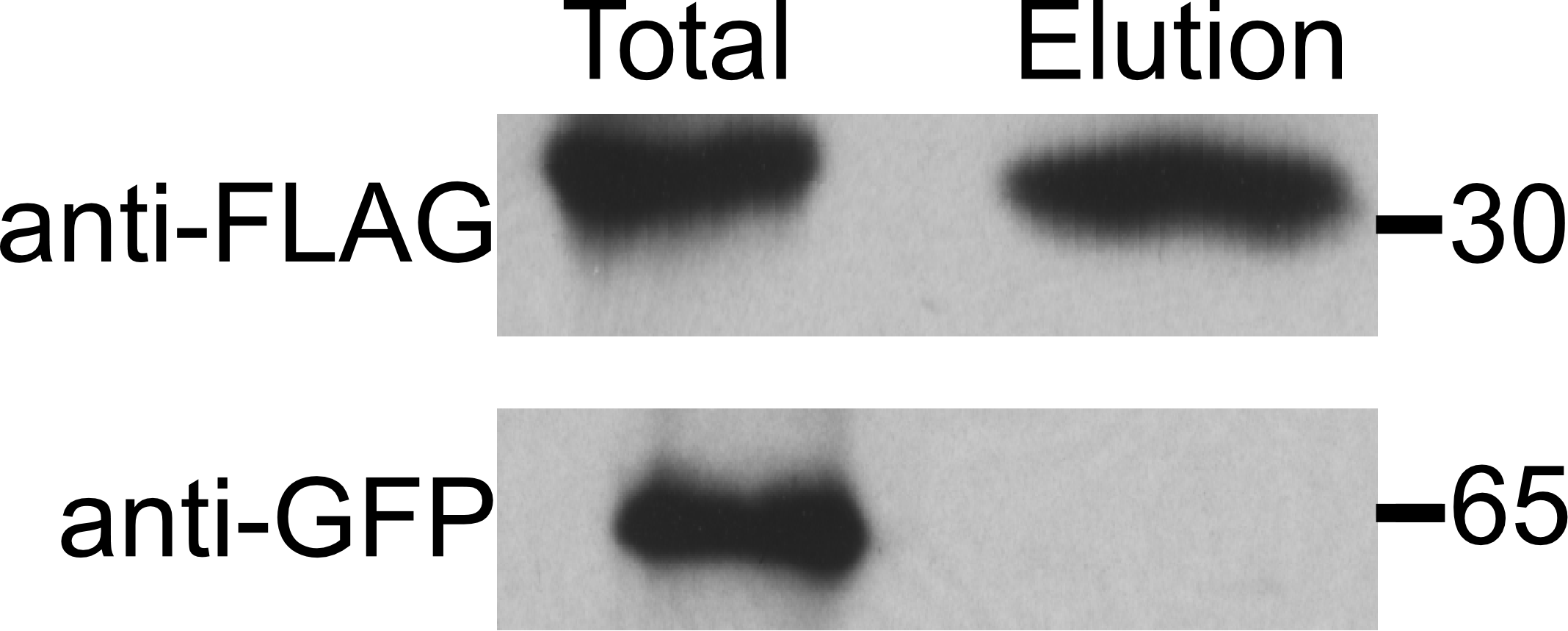

Supplement: Figure S2 — Co-immunoprecipitation assays. Western blots of total proteins isolated from transformant DCN22 (expressing the CAP1− ΔACB-GFP and MAC1 CT-3×FLAG constructs) and proteins eluted from anti-FLAG M2 beads were detected with an anti-FLAG or anti-GFP antibodies. ACB: Putative N-terminus AC-binding domain of Cap1. (TIF) [file ppat.1002911.s002.tif]

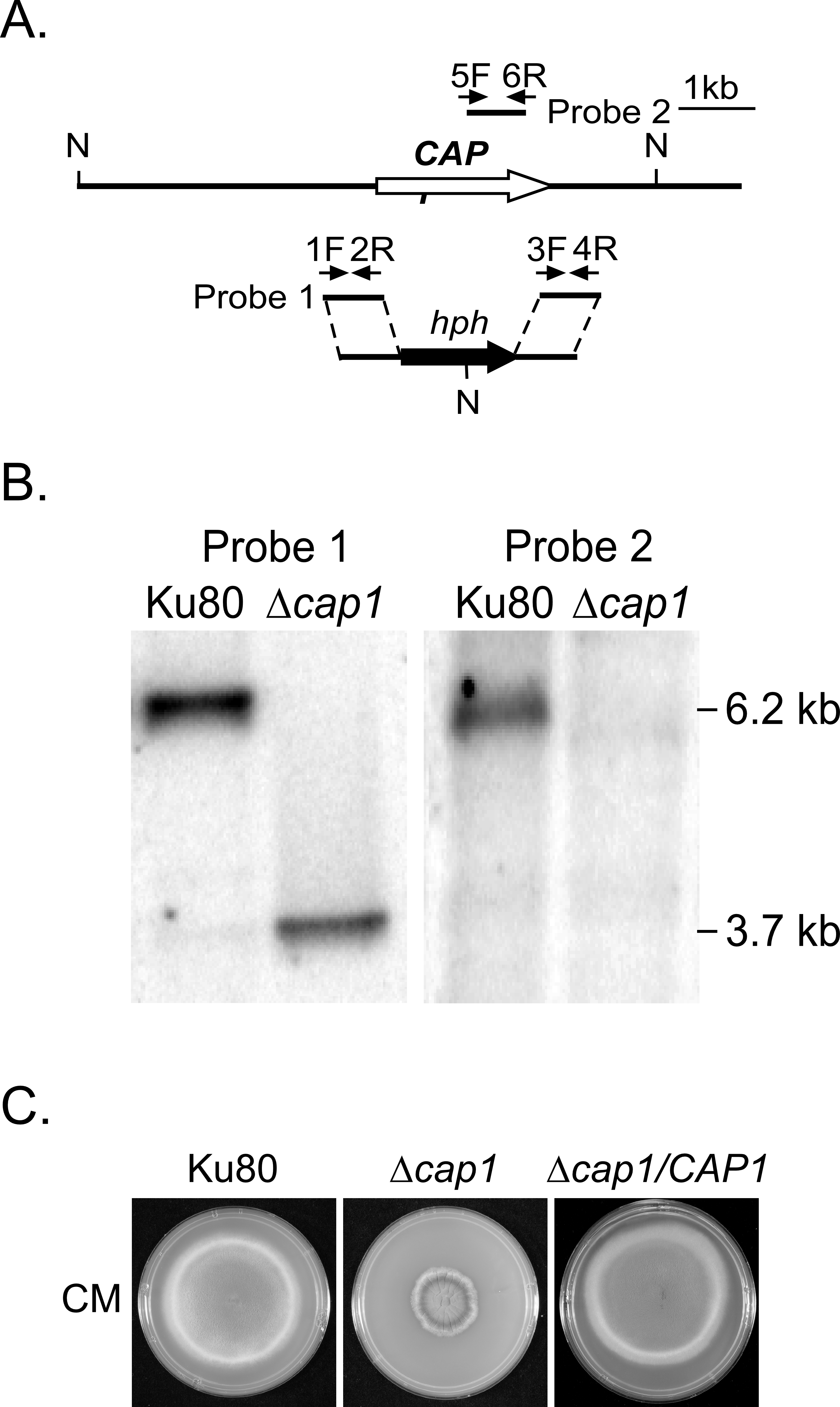

Supplement: Figure S3 — Generation of the CAP1 gene replacement construct and mutant. A. The genomic region of the CAP1 gene and PCR fragments used for constructing the gene replacement mutants and hybridization. PCR primers are marked with small arrows. N, NcoI. B. Blots of NcoI-digested genomic DNA of Ku80 and the Δcap1mutant were hybridized with probe 1 and probe 2 (see Fig. S3A). Probe 1 detected a 6.2-kb band in Ku80 but a 3.7-kb band in the Δcap1 mutant. When hybridized with probe 2, only strain Ku80 had the 6.2-kb band. C. One-week-old CM cultures of Ku80, Δcap1 mutant, and Δcap1/CAP1 transformant. (TIF) [file ppat.1002911.s003.tif]

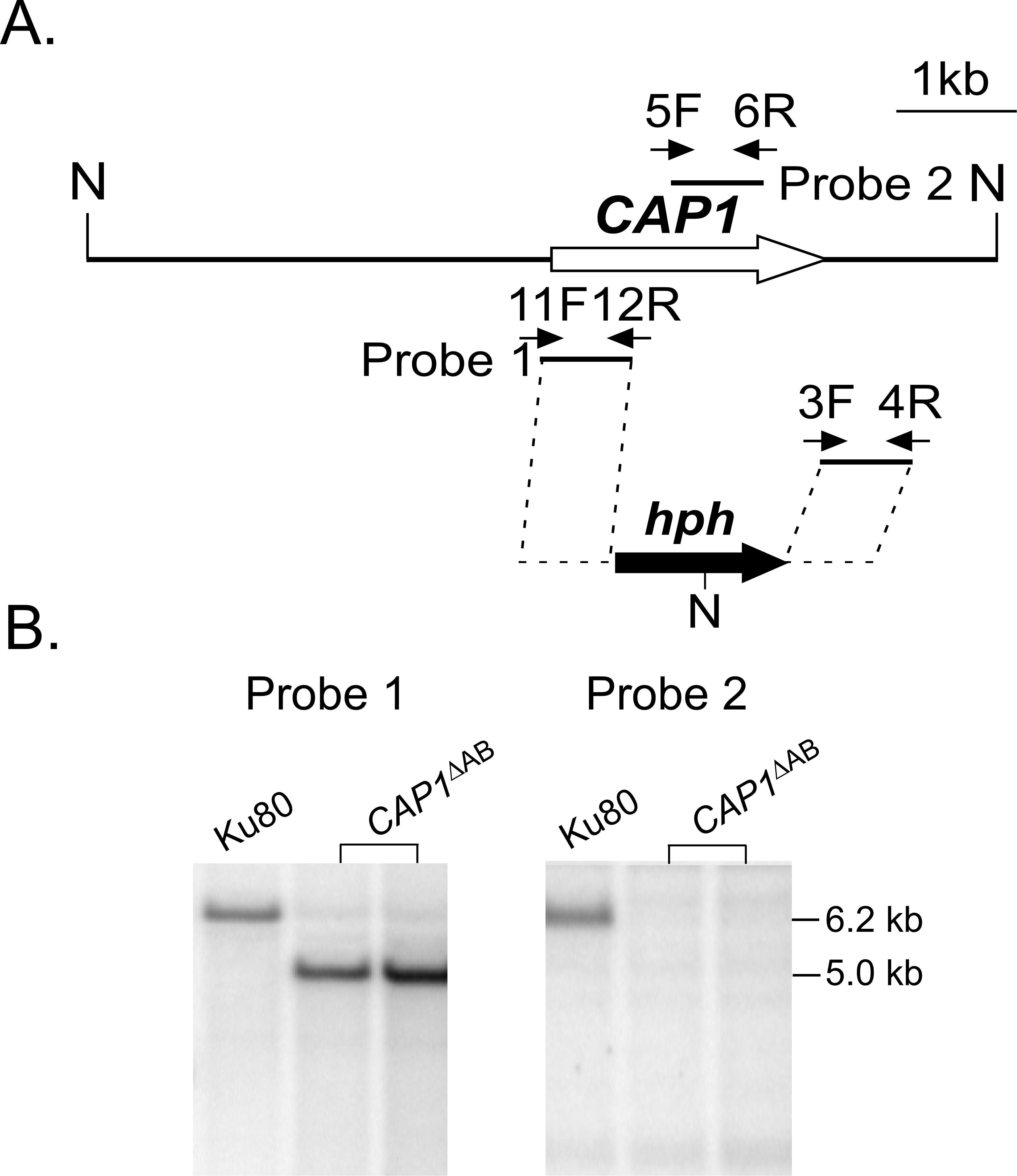

Supplement: Figure S4 — Generation of the CAP1 ΔAB mutant. A. Small arrows mark the PCR primers used to amplify genomic fragments for generating the CAP1 ΔAB allele and probes for hybridization. N, NcoI. B. Blots of NcoI-digested genomic DNA of Ku80 and CAP1 ΔAB mutants were hybridized with probe 1 and probe 2. Probe 1 detected a 6.2-kb band in Ku80 but a 5.0-kb band in the CAP1 ΔAB mutant. When hybridized with probe 2, only strain Ku80 had the 6.2-kb band. (TIF) [file ppat.1002911.s004.tif]

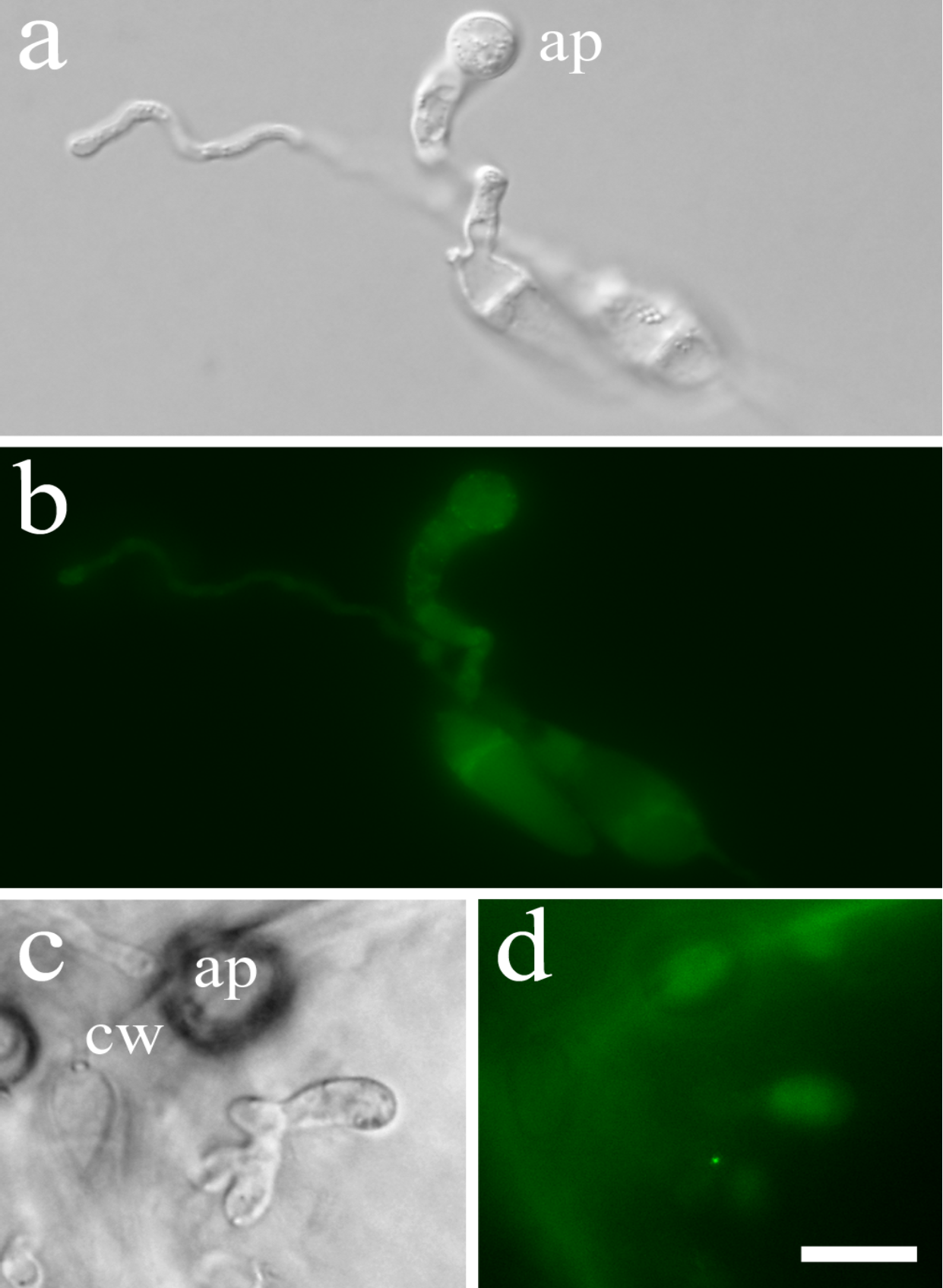

Supplement: Figure S5 — Localization of Cap1ΔP2-GFP in germ tubes, young appressoria, and invasive hyphae. a–b) Germ tubes and appressoria of the CAP1 ΔP2-GFP transformant examined by DIC and epifluoresence microscopy. c–d) Invasive hyphae of the CAP1 ΔP2-GFP transformant formed inside onion epidermal cells. ap: appressorium; cw: onion cell wall. Bar = 10 µm. (TIF) [file ppat.1002911.s005.tif]

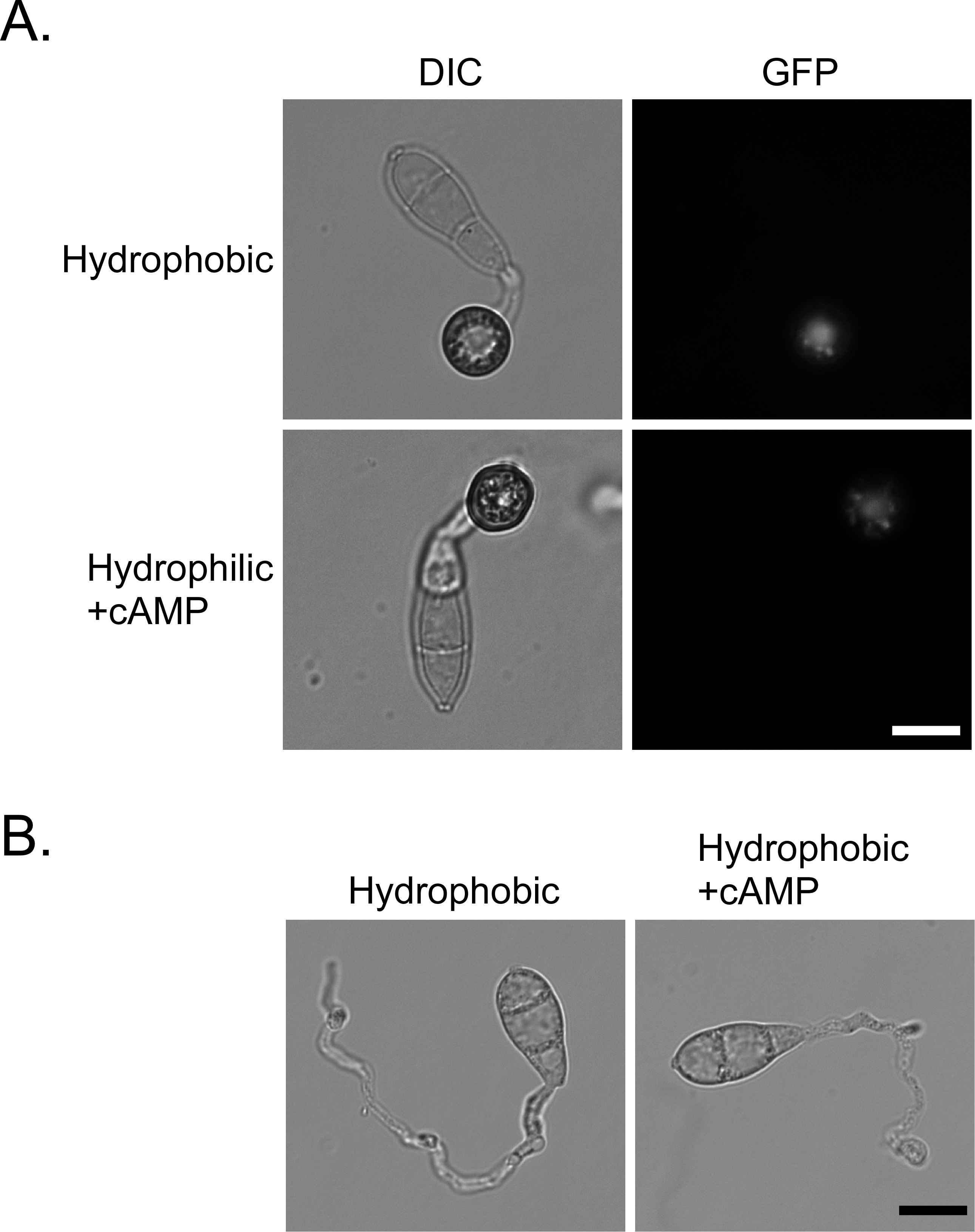

Supplement: Figure S6 — A. Localization of GAS2-GFP in appressoria formed on hydrophobic (upper panel) and hydrophilic surfaces in the presence of 5 mM cAMP. B. Appressorial formation assays with the CAP1 ΔAB/Δpmk1 transformant on hydrophobic surfaces with or without exogenous cAMP. (TIF) [file ppat.1002911.s006.tif]
