# Supplementary material for: The Cyclase-Associated Protein Cap1 Is Important for Proper Regulation of Infection-Related Morphogenesis in Magnaporthe oryzae
Source: PLoS Pathog. 2012 Sep 6;8(9):e1002911. doi: 10.1371/journal.ppat.1002911 (PMC3435248; doi:10.1371/journal.ppat.1002911)
Supplement: Table S1 — PCR primers used in this study. (DOCX) [file ppat.1002911.s007.docx]

**Table S1.** PCR primers used in this study

| **Name** | **Sequence (5’-3’)** | **Applications** |
| --- | --- | --- |
| 1F | tgcttattggcgactgatattgcatc | *CAP1* knockout |
| 2R | atatggccggccgtgtcggtacgggttattgaggt |  |
| 3F | aattggcgcgccagtcctatagttctgcctgttctctg |  |
| 4R | cagcatcaacttcaacatcgtctcca |  |
| NF | gcaacggactgtcttggaac |  |
| NR | cgagtcaatgatgagcgaaa |  |
| CF | atcgattgcggccgcgtcaaaggcattgtgctgaactgattcag |  |
| CR | tatacgctcgagaatcatctactgtagacaagacgcaccatg |  |
| FlagF | cactatagggcgaattgggtactcaaattggttgtcaaaggcattgtgctgaactgattcag | *CAP1*-3xFLAG |
| FlagR | ctttataatcaccgtcatggtctttgtagtcaccagcgtgctcaacaatctcgttcac | *CAP1*-3xFLAG |
| GFPR | caccccggtgaacagctcctcgcccttgctcacaccagcgtgctcaacaatctcgttcac | *CAP1*-GFP |
| T300 | ccagcagtagacacttggaa |  |
| ACB3F | aattggcgcgcccatggatagcgctcagttgtttggca | *CAP1*^∆ACB^ |
| ACB4R | ggctctggctgctgctcaaagttctc | *CAP1*^∆ACB^ |
| AB1F | gttatcaaagggcttccaactacagcg | *CAP1*^∆AB^ |
| AB2R | cgctgtagttggaagccctttgataac | *CAP1*^∆AB^ |
| ACR1 | catattttggcggctagtcgtggttggcagtc | *CAP1*^∆ACB^-GFP |
| ACF2 | gactgccaaccacgactagccgccaaaatatgaccgccgaagccgtgatggttct | *CAP1*^∆ACB^-GFP |
| ABFR | ctttataatcaccgtcatggtctttgtagtcttgtttccgtcaagctccttc | *CAP1*^∆AB^-GFP |
| ABGR | caccccggtgaacagctcctcgcccttgctcacttgtttccgtcaagctccttc | *CAP1*^∆AB^-GFP |
| P1R1 | tgactgcatgctcttcataacctgttggac | *CAP1*^∆P1^-GFP |
| P1F2 | gtccaacaggttatgaagagcatgcagtcactaaagatcaaggagcaaggggcagcgtc | *CAP1*^∆P1^-GFP |
| P2R1 | gacgaaacagacccctcccgttcagga | *CAP1*^∆P2^-GFP |
| P2R2 | tcctgaacgggaggggtctgtttcgtcgaaggagcttgacggaaacaa | *CAP1*^∆P2^-GFP |
| Mac1-FLF | tttcgtaggaacccaatcttcaaaatgtcggagattcagagatgcctggagacttacca | *MAC1*^CT^-3xFLAG |
| Mac1-FLR | cagaggagcctgaatgttgagtggaatgatttacttgtcatcgtcatccttgtaatcgatatcatgatctttataatcaccgtcatggtctttgtagtcagatgcttgctggccatcctcaatcg | *MAC1*^CT^-3xFLAG |
| MAC-CNF | gagctacggtcactcagca |  |
| MAC-CNR | ccatcctcaatcgcatcaa |  |
| CAP1-YF | atactagctagcatggctaccaacaatatgcataacctaact | Two-hybrid |
| CAP1-YR | atactagaattcaccagcgtgctcaacaatctcgttc | Two-hybrid |
| MAC1-YF | acttgagaattcatggagattcagagatgcctggagacttaccag | Two-hybrid |
| MAC1-YR | acttgagtcgacagatgcttgctggccatcctcaatc | Two-hybrid |
| CBFR | gctcaccatcgtggcgatggagcgaccagcgtgctcaacaatctcgttcac | BiFC |
| MBR | gttcgggatcttgcaggccgggcgagatgcttgctggccatcctcaatcg | BiFC |
| RAS2QF | acaaggcaagatcgagacatcgg |  |
| RAS2QR | gcctcagcatcgctatcgttga |  |
| MACQF | caccgagaccagaagagaagatgac |  |
| MACQR | gtagagcgatgtggtactcgagac |  |
| LifeActF | tttcgtaggaacccaatcttcaaaatgggtgtcgcagatttgatcaagaaattcgaaagcatctcaaaggaagaaggcggaggcggcggaggcggaggcggaggcgtgagcaagggcgaggagctgttcaccggggtggtg | LifeAct-GFP |
| LifeActR | caccaccccggtgaacagctcctcgcccttgctcacgcctccgcctccgcctccgccgcctccgccttcttcctttgagatgctttcgaatttcttgatcaaatctgcgacacccattttgaagattgggttcctacgaaa | LifeAct-GFP |
